# Habitat and lifestyle affect the spatial dynamics of prokaryotic communities along a river–estuary–sea continuum

**DOI:** 10.1002/mlf2.70017

**Published:** 2025-06-20

**Authors:** Jiao Liu, Peng Yao, Jinmei Liu, Gaoyang Ren, Xiao‐Hua Zhang, Jiwen Liu

**Affiliations:** ^1^ College of Marine Life Sciences, and Frontiers Science Center for Deep Ocean Multispheres and Earth System Ocean University of China Qingdao China; ^2^ Laboratory for Marine Ecology and Environmental Science Qingdao National Laboratory for Marine Science and Technology Qingdao China; ^3^ Key Laboratory of Marine Chemistry Theory and Technology, Ministry of Education Ocean University of China Qingdao China; ^4^ Key Laboratory of Evolution & Marine Biodiversity (Ministry of Education) and Institute of Evolution & Marine Biodiversity Ocean University of China Qingdao China

**Keywords:** Changjiang River, community assembly, habitat, microbial community, river–estuary–sea continuum

## Abstract

Microbial biogeography and its controlling mechanisms are central themes in microbial ecology. However, we still lack a comprehensive understanding of how habitats and lifestyles affect microbial biogeography across complex environmental gradients. In this study, we investigated the planktonic (including free‐living [FL] and particle‐associated [PA] lifestyles) and benthic prokaryotic communities along a river–estuary–sea continuum of the Changjiang River to explore their distinct spatial dynamics. We observed greater community variability across spatial distances than between habitat and lifestyle types. Spatial variations were evident in FL, PA, and benthic communities, with the highest turnover rates observed in benthic communities, followed by PA, and the lowest turnover rates observed in FL. The replacement effect dominated PA and benthic community variations, whereas the richness effect was more significant in FL communities. Microbial assembly was primarily governed by homogeneous selection and dispersal limitation regardless of habitats/lifestyles, with their ratios decreasing as the spatial distance increased, particularly in the FL fraction. Dispersal limitation had a stronger effect on benthic communities compared to planktonic communities. While heterogeneous selection generally played a minor role, its influence became more pronounced over larger spatial distances and with increasing salinity differences. Finally, we showed that abiotic and biotic factors individually exerted a greater influence on PA communities, whereas their interactions had a stronger effect on FL communities. Our results revealed complex spatial dynamics and assembly mechanisms among microorganisms across different habitats and lifestyles, providing insights into the spatial scaling of community assembly across complex environmental gradients.

## Impact statement

The river–estuary–sea continuum presents a natural environmental gradient that fosters rich microbial communities. However, our understanding of microbial community succession patterns and underlying ecological mechanisms remains limited. In this study, we observed significant changes in prokaryotic communities along the river–estuary–sea continuum. We found that spatial effect was more important in structuring community variations than that exerted by habitat or lifestyle types. Increasing spatial distance led to a greater effect of dispersal limitation on community assembly, and heterogeneous selection intensified its role at higher salinity differences. These findings provide new insights into the assembly processes of microbial communities in natural ecosystems, characterized by high spatial connectivity and environmental heterogeneity.

## INTRODUCTION

Microorganisms with highly diverse taxonomic and functional guilds are critical to ecosystem functioning and service^1^. Changes in microbial community composition can affect ecosystem function, which highlights the importance of unraveling factors governing community variations. The process of microbial community assembly is constrained under the niche and neutral theory^2^. Ecological niche theory suggests that both deterministic abiotic and biotic factors shape microbial communities, influencing their habitat preferences and adaptive strategies^3^, ^4^. The neutral theory assumes that microbial species share equal ecological niches and that community assembly is mainly shaped by stochastic events, such as birth, death, migration, and dispersal^5^, ^6^. Stochastic processes result in unpredictable compositional changes, while deterministic processes influence the fitness of microbial communities, thus affecting species composition and abundance^7^, ^8^. Both processes affect the role of microbial communities in biogeochemical cycles^9^, ^10^. To predict the response of microbial community and function under changing environments, it is imperative to assess the balance of these processes across broad and distinctive environments.

A growing body of studies has investigated the ecological mechanisms underpinning microbial community assembly, many of which are conducted using limited temporal and spatial scales, which can restrict our understanding of the dynamics of community assembly with environmental changes. In rivers covering large spatial scales, progressive microbial community changes along the river path have been observed. For example, using samples spanning hundreds to thousands of kilometers, spatial community dynamics were investigated in the La Romaine River in Canada^11^, the Changjiang and Nu River in China^12^, ^13^, and the River Thames in UK^14^. At the same time, other efforts were devoted to exploring changes of microbial populations in estuaries and/or coastal areas, reporting distinct community compositions between freshwater and seawater^15^, ^16^. The river–estuary–sea continuum encompasses natural salinity and nutrient gradients across large spatial distances^16^, ^17^. Understanding the large‐scale spatial distribution pattern of microbial communities is crucial for comprehending how these communities respond to multiple environmental factors along this continuum. However, few studies included spatially continuous samples covering river flow, estuaries, and coastal ocean, which limits the exploration of the spatial changing pattern of microbial community assembly.

Microbial communities include those occupying different habitats (e.g., water and sediment) and adopting different lifestyles (e.g., free‐living [FL] and particle‐associated [PA]). An important question that remains unknown is whether the spatial assembly pattern of microbial communities is affected by habitat/lifestyle types. Water and sediments have contrasting hydrological and physicochemical differences, and it is well known that microbial communities between these two systems are significantly distinct^18^. Compared to water, microbes in sediments may have relatively low dispersal rates and high resistance to environmental disturbances^19^, ^20^, which may result in their distinct community spatial dynamics. In water, suspended particulate matter creates unique microhabitats for PA microbes, distinct from the living condition of FL microbes. Microbes attached to particles may be subject to restricted dispersal, thereby experiencing stronger dispersal limitation^21^. Considering these differences, it is crucial to delve deeper into the assembly patterns of microbial communities across various habitats and lifestyles. In large riverine continuum systems, the diverse channels, strong environmental gradients, and various hydraulic constructions disturb the microbial communities living there. However, a direct comparison of ecological mechanisms governing the assembly of these different microbial fractions is lacking, which limits our understanding of their distinct spatial dynamics and ecological functions.

In this study, we analyzed the geographical pattern and assembly process of prokaryotic communities using samples over a large spatial scale along a river–estuary–sea continuum of the Changjiang River, China. Parallel collection of water and sediment samples, and FL and PA fractions from a single site allow us to answer the following questions: (1) How does microbial community develop along the river flow? (2) Do the prokaryotic communities show spatial and habitat variability? (3) How do prokaryotic communities assemble in this large river–estuary–sea system in terms of the effect of environmental gradients and habitat/lifestyle? Our results will expand the knowledge of large‐scale microbial spatial patterns from river to ocean and contribute toward understanding the biogeography of microbial community assembly.

## RESULTS

### Prokaryotic abundance and diversity along the spatial gradient

**Figure 1:**
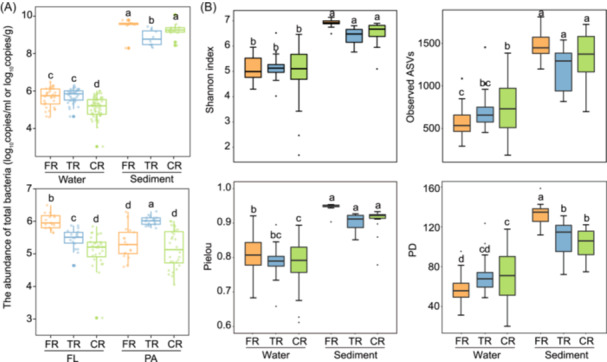
Bacterial abundance and alpha diversity of prokaryotic communities. (A) Bacterial abundance (log_10_ copies/ml or log_10_ copies/g) in different regions (freshwater region [FR] vs. transition region [TR] vs. coastal region [CR]), different habitats (water vs. sediment), and different lifestyles (free‐living [FL] vs. particle‐associated [PA]). Lower‐case letters indicate significant differences (Wilcoxon's rank‐sum test). (B) Alpha diversity indices in different regions (FR vs. TR vs. CR) and different habitats (water vs. sediment). Lower‐case letters indicate significant differences (ANOVA, Tukey's post hoc test). ASVs, amplicon sequence variants; PD, phylogenetic diversity.

Planktonic and benthic prokaryotic communities covering a large spatial scale (~1900 km, a conservative estimate based on linear distance) along the river–estuary–sea continuum of the Changjiang River were analyzed (Table S1). Quantification analysis showed a higher abundance of bacteria in sediment (from 1.96 × 10^8^ to 1.20 × 10^10^ copies/g) than in water (from 1.08 × 10^3^ to 3.09 × 10^6^ copies/ml) (*p* < 0.05, Wilcoxon rank‐sum test) (Figure 1A). Bacterial abundance was generally higher in the freshwater region (FR) compared to the coastal region (CR), although a significant difference was observed only in water (*p* < 0.05) (Figure 1A). In water, FL bacteria (1.08 × 10^3^ to 3.09 × 10^6^ copies/ml) were generally more abundant than PA bacteria (1.13 × 10^4^ to 2.01 × 10^6^ copies/ml) (*p* < 0.05) (Figure 1A), with the exception of a higher PA bacterial abundance in the transition region (TR) (Figure 1A). FL bacteria decreased in abundance from FR to CR, whereas PA bacteria showed a pattern of an initial increase, followed by a subsequent decrease.

The species diversity of microbial communities remained generally consistent among the sampling regions, except for higher values of the Pielou index in FR (Tukey's, *p* > 0.05). Nonetheless, distinct spatial patterns of species diversity emerged between water and sediments. In the water, while the Shannon index remained relatively stable across regions, the observed amplicon sequence variants (ASVs) and phylogenetic diversity (PD) indices increased toward the CR (Figure 1B). The spatial variation trends of both size fractions aligned with those of the total community, though the PA fraction displayed greater diversity than its FL counterpart (Figure S1). In sediments, the values of Shannon, observed ASVs, and Pielou indices were the lowest in TR and were marginally different among regions (Figure 1B).

### Community composition and occupancy pattern

**Figure 2:**
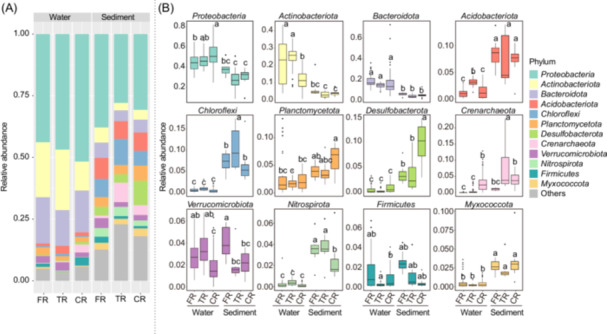
Prokaryotic community compositions across samples. (A) Phylum‐level compositions. (B) Shifts in the top 12 abundant phyla. Lower‐case letters indicate significant differences (ANOVA, Tukey's post hoc test).

A total of 31,439 ASVs were generated and taxonomically classified into 84 phyla and 2026 genera. *Proteobacteria*, *Bacteroidota,* and *Actinobacteriota* were predominant, but their abundance varied between habitat types and along the spatial continuum (Figure 2A). Across all regions, *Bacteroidota* and *Actinobacteriota* were more abundant in water, while *Acidobacteriota*, *Chloroflexi*, *Desulfobacterota*, *Crenarchaeota,* and *Planctomycetota,* encompassing generally lower proportions, were enriched in sediments (Figures 2A and S2). In water, the TR communities were more similar to FR than to CR. There appeared to be a slight decline in *Bacteroidota* and *Firmicutes*, along with a slight increase in *Proteobacteria*, *Actinobacteriota*, and *Acidobacteriota* from FR to TR, although most of these changes were not significant (Figure 2). As the degree of mixing with freshwater and seawater increased, *Actinobacteriota* significantly decreased in CR, while *Crenarchaeota* and *Firmicutes* experienced a notable increase (*p* < 0.05) (Figure 2). The two size fractions (FL and PA) showed generally similar spatial patterns as those of the overall community, although the relative abundance of specific taxa, such as *Actinobacteriota* and *Planctomycetota*, varied between them (Figure S3). The benthic communities, however, showed distinct spatial variations, with the compositions being more similar between TR and CR. This was mirrored by a decreasing trend in the relative abundance for most freshwater taxa as freshwater meets seawater (Figure 2B).

**Figure 3:**
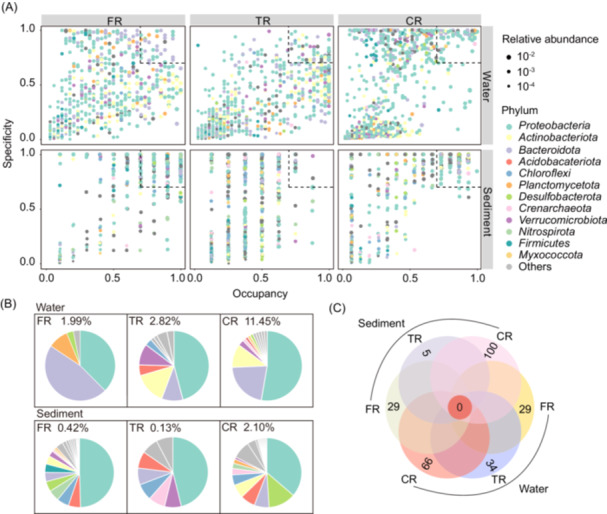
Prevalence and specificity of ASV distributions. (A) SPEC‐OCCU plots showing the 1000 most abundant ASVs in different regions. The *x*‐axis represents occupancy, indicating the distribution of ASVs across all sampling sites in each habitat; the y‐axis represents specificity, indicating habitat preference. The dotted box shows specialist ASVs with specificity and occupancy greater than or equal to 0.7. (B) Relative abundance and taxonomic classification of specialist ASVs in different samples. (C) Venn diagram showing the numbers of unique and shared ASVs among groups.

To examine the uniqueness and commonness of ASVs across samples, specificity and occupancy were calculated for each ASV and were projected onto a scatter plot (Figure 3A). ASVs from the FR planktonic community showed highly variable specificity and occupancy. The majority of them showed low specificity (shared with TR and CR) and low occupancy (few were present in all FR stations). This phenomenon was more pronounced for ASVs in TR and CR, suggesting that most of the taxa in these two regions were shared with the other regions, although they showed distributional specificity between stations within a region. Notably, ASVs from CR showed obvious polarization, with some high‐specificity ASVs and some low‐occupancy ASVs, suggesting that there was a high degree of variability in ASVs among CR stations (Figure 3A). The specificity and occupancy of ASVs in sediments showed marked differences among regions. Specifically, CR sediments harbored numerous ASVs with both low specificity and low occupancy. This indicated that these ASVs, while shared with the other spatial regions, were limited to only a few CR sites, suggesting relatively stronger niche heterogeneity in the coastal ocean.

To identify ASVs specific to each sampling type, we chose ASVs with specificity and occupancy values ≥ 0.7 (indicated by dotted boxes in Figure 3A). This criterion ensures that the selected ASVs are both specific to a particular type of samples and common across stations within that type. The number of ASV specialists varied among the spatial regions, with freshwater planktonic ASVs showing lower specialist diversity compared to TR and CR. *Proteobacteria* dominated the specialist ASVs in all regions, particularly in TR and CR (Figure 3B). In FR water, *Bacteroidota* were more dominant specialists, followed by *Proteobacteria* and *Planctomycetota*. Despite *Actinobacteriota* being the dominant group in FR water, no specialists were identified within this phylum. On the contrary, several ASVs from *Actinobacteriota* and *Verrucomicrobiota* emerged as specialists in the downstream TR and CR water. Similar to the planktonic community, *Proteobacteria* were the primary specialists across all regions in the benthic community. From FR to CR, the taxonomic diversity of benthic specialists displayed a spatial trend of first decreasing and then increasing. Specifically, FR displayed the highest diversity of specialist taxa, followed by the CR, while TR showed the lowest diversity. Although certain specialist taxa were found in multiple regions, no specialist ASVs were shared across these regions (Figure 3C).

We next examined the specificity and occupancy of prokaryotic taxa inhabiting different habitat types (i.e., FL, PA, and sediment). The ASVs from each habitat showed highly varying occupancy, with only a few ASVs present in all samples (Figure S4A). This was likely attributable to the significant spatial turnover of prokaryotic taxa along the river–estuary–sea continuum regardless of habitats. Species specialization varied among lifestyles, with FL specialists dominated by *Proteobacteria* and *Actinobacteriota* and PA specialists dominated by *Bacteroidota*. In sediment, no specialist ASVs were detected (Figure S4B).

### Biogeographic pattern of prokaryotic communities

**Figure 4:**
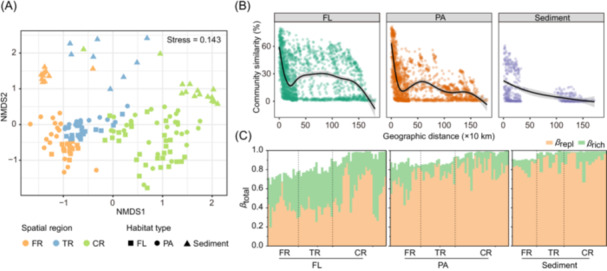
Community comparison, distance–decay pattern, and *β*‐diversity partitioning pattern at the ASV level. (A) Nonmetric multidimensional scaling ordination of community based on the Bray–Curtis dissimilarity. (B) Pairwise relationships between Bray–Curtis dissimilarities and geographic distances for water (FL and PA) and sediment samples. (C) *β*‐diversity partitioning patterns in different habitats. *β*_total_, total *β*‐diversity; *β*_repl_, species replacement; *β*_rich_, richness difference.

Prokaryotic community compositions differed significantly among spatial regions (Permutational multivariate analysis of variance test (PERMANOVA), *R* ^2^ = 0.21, *p* = 0.001), habitats (*R* ^2^ = 0.10, *p* = 0.001), and lifestyles (*R* ^2^ = 0.03, *p* = 0.001), with the communities being more variable among spatial regions. Nonmetric multidimensional scaling (NMDS) ordination revealed clear compositional shifts in all communities from FR to CR (Figure 4A and Table S2) and the spatial variability was the highest in FL communities, moderate in benthic communities, and the lowest in PA communities (Table S3). Furthermore, FL and PA communities showed different levels of pairwise compositional variations within each sampling site along the river flow, with the lowest level seen in TR (Figure S5).

The similarity of microbial communities among geographically proximate samples was evident in a clear distance–decay relationship, characterized by increasing community dissimilarity with greater geographic distance (Figure S6). This pattern was seen in both water (FL: *R* ^2^ = 0.046, *p* < 0.001; PA: *R* ^2^ = 0.165, *p* < 0.001) and sediment (*R* ^2^ = 0.261, *p* < 0.001) samples, and was more pronounced in the latter. In water, similarities of the PA community decreased from 82% to 21% over a distance of ~1500 km, whereas those of the FL community showed a comparatively modest change, with similarities shifting by only ~45%. At distances exceeding ~1300 km, the FL community underwent a sharp decline in similarities from 60% to 5% (Figure 4B). Furthermore, the distance–decay pattern showed notable variations within each specific spatial region (Figure S6). Spatial changes in the planktonic community were most pronounced in TR, while the weakest changes were observed in FR. The shifts within TR were more accentuated in the PA than the FL fraction. Conversely, the benthic community showed substantial turnover in FR.

To analyze the spatial community variation contributed by different components, the overall *β‐*diversity was partitioned into species replacement (*β*_repl_) and richness difference (*β*_rich_). The most upstream sampling point (ZT) was used as the base point, which was compared with each downstream sampling point. Along the river flow, the *β*‐diversity increased and approached one in the CR (Figure 4C), indicating almost no shared taxa between the locations. Species replacement dominated the community shifts across all habitat types, but were more pronounced in the PA and benthic community. Species replacement was also prevalent in the coastal FL community, but its contribution was equal to or surpassed by the richness effect in FR and TR. This indicated a significant increase in the frequency of species replacement in the FL community upon entering seawater (*p* < 0.05, Wilcoxon's rank‐sum test). Overall, replacement effects were greater for benthic taxa than for planktonic taxa (*p* < 0.05, Wilcoxon's rank‐sum test), while richness effects were greater for FL than for PA and benthic taxa (*p* < 0.05, Wilcoxon rank‐sum test) (Figure 4C).

We then examined the *β*‐diversity partition patterns of the top abundant microbial phyla to explore their respective contributions to the meta‐community *β*‐diversity (Figure S7). The contribution of different groups differed between habitat types. In the FL fraction, *Bacteroidota*, *Verrucomicrobiota*, and *Actinobacteriota* were mainly shaped by taxonomic replacement, whereas *Proteobacteria* and *Crenarchaeota* were more influenced by richness effects. In the PA community, *Proteobacteria*, *Planctomycetota*, *Bacteroidota*, and *Actinobacteriota* were major contributors to replacement effects, whereas *Firmicutes* contributed more to richness effects. FL *Proteobacteria* and *Actinobacteriota* were less influenced by species replacement than PA and benthic taxa, but were greatly impacted by richness effects. Changes in *Crenarchaeota* fluctuated along the river flow, with alternating replacement and richness effects, while in CR, richness effects predominantly dominated.

### Ecological progress shaping geographic patterns

**Figure 5:**
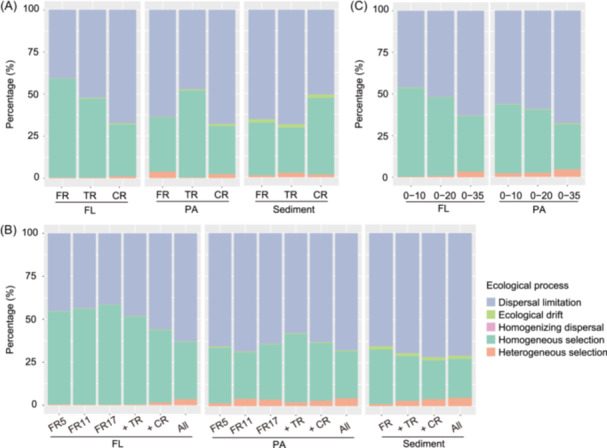
Assembly mechanisms of prokaryotic communities by null model analysis. (A−C) The assembly mechanisms compared among different regions (A), geographical distances (B), and salinity ranges (C). FR5, FR11, and FR17 represent the initial 5, 11, and 17 sampling sites in the FR, respectively. Site 5 marks the boundary between the upper and middle reaches, site 11 delineates the boundary between the middle and lower reaches, and site 17 represents the final station of the river section, marking the transition to the estuary. The plus sign indicates a cumulative effect. For example, “+ TR” means samples from a previous group are combined with those from the TR for analysis.

The proportional contribution of different community assembly processes was quantified using a bin‐based null model analysis (iCAMP). The outcomes revealed that homogeneous selection and dispersal limitation were the dominant processes, accounting for 22.5%–58.7% and 40.7%–71.8% of the community variation across all samples, respectively (Figure 5A). Their relative contributions differed between habitats and lifestyles. Dispersal limitation had a greater impact on PA and benthic taxa, while FL taxa were more influenced by homogeneous selection, especially in FR.

Because the sampling sites (from freshwater to seawater) spanned a wide range of spaces with strong environmental gradients, we examined changes in different ecological mechanisms with spatial distance. For all communities, the proportion of heterogeneous selection slightly increased with spatial distance, with a stronger effect on benthic and PA taxa than on FL taxa (Figure 5B). Furthermore, the relative contribution of dispersal limitation to FL and PA taxa decreased with increasing spatial extent within FR, but showed an increasing trend when extended to CR and TR (Figure 5B). Because salinity is the crucial factor changed from freshwater to seawater, we evaluated the change of ecological processes under different salinity gradients (Figure 5C). Among the three salinity groups, the impact of heterogeneous selection was minimal (0.3%−2.1%) under the 0−10 salinity gradient, increased slightly to 0.8%−2.5% under 0−20, and further increased to 3.4%−4.5% under 0−35. This shift in heterogeneous selection was concurrent with a rising trend in dispersal limitation.

### The effect of abiotic and biotic variables

**Figure 6:**
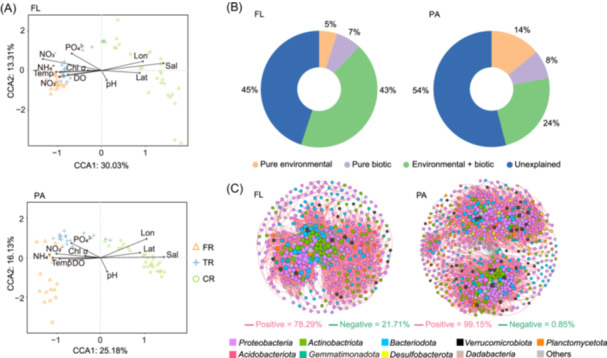
Effect of environmental factors and biotic interactions on prokaryotic communities. (A) The relationship between prokaryotic community structure at the ASV level and environmental variables illustrated by CCA analysis. Environmental factors include the longitude (Lon), latitude (Lat), salinity (Sal), temperature (Temp), pH, dissolved oxygen (DO), Chl *α*, NH_4_^+^, NO_3_^−^, NO_2_^−^, and PO_4_^3−^. (B) Variation partitioning of biotic and environmental variables. The percentages are given for the relative contribution of each component to the community variation, as indicated by different colors. (C) Co‐occurring network of planktonic communities based on correlation analyses.

Significant environmental heterogeneity was observed across regions (PERMANOVA, *R* ^2^ = 0.69, *p* = 0.001), with the greatest differences between FR and CR (FR vs. TR, *R* ^2^ = 0.10, *p* = 0.006; FR vs. CR, *R* ^2^ = 0.62, *p* = 0.003; TR vs. CR, *R* ^2^ = 0.61, *p* = 0.003), as expected. According to the canonical correspondence analysis (CCA) and Mantel tests, salinity, longitude, latitude, SiO_3_^2−^, NO_3_^−^, temperature, NH_4_^+^, PO_4_^3−^, dissolved oxygen (DO), NO_2_^−^, and Chl *α* significantly correlated with both FL and PA communities, while pH was correlated only with PA communities (Figure 6A, Table S4). Partial Mantel tests showed that the significance of all environmental factors remained, except for Chl *α* for the PA community and NO_2_^−^ for the FL community, when spatial distance was controlled.

We used interaction indices obtained through network analysis to represent biotic variables. The results indicated that the pure effect of biotic factors (7%) was more important in explaining FL communities compared to environmental factors (5%). In contrast, for PA communities, the pure effect of biotic factors (8%) had less explanatory power than environmental factors (14%) (Figure 6B). However, both the pure effect of environmental and biotic factors contributed more to variations of the PA than FL communities. The joint effect of biotic and environmental factors was more important in explaining the FL (43%) compared to the PA (24%) community variances.

Based on the correlation network, the role of biotic interactions in shaping communities was further explored. The FL and PA co‐occurrence network consisted of 404 nodes linked by 7689 edges and 656 nodes linked by 11,583 edges, respectively (Figure 6C; Table S5). This indicated that the microbial communities had more potential interactions in the PA than in the FL. The community network increased in complexity from FL to PA fractions, and from freshwater to coastal regions, as evidenced by the total number of nodes, edges, and the average degree (Table S6). However, network modularity was higher in the freshwater area, indicating higher functional diversity. Interestingly, the PA network in general showed higher modularity than the FL network, whereas in each regional subnetwork, the modularity was higher in the FL network. Robustness analysis using species extinction simulation showed that the PA communities in TR and CR showed higher stability than the FL communities (Figure S8). In both lifestyles, the TR community had a more stable network structure compared to the other two regions.

## DISCUSSION

### Microbial diversity and spatial distribution patterns

Rivers serve as conduits for both materials and biodiversity, facilitating the coalescence of microbial communities across diverse environmental conditions and their dispersal downstream^22^. As tributaries introduce their unique microbial communities, the downstream accumulation of microbial diversity is enhanced^23^. Consistently, we observed that the prokaryotic community in water generally increased in richness from upstream to downstream along the Changjiang River, similar to those found in the Nu River^13^ and in the Three Gorges Reservoir^24^. In contrast to diversity, the abundance of bacteria showed an opposite trend, which may be due to habitat reduction caused by increased environmental stresses such as pollution emissions in downstream. We further observed increased richness but declined abundance in seawater compared to freshwater, unlike a previous study in the northern Gulf of Mexico^25^. The particular environment of the Changjiang River Basin might tend to select some specific bacterial lineages that are phylogenetically close to each other and maintain them at high abundance levels. In the coastal ocean, the convergence of microbes from diverse sources such as freshwater discharge and distinct water masses may contribute to a high diversity. However, growth of these microbes in coastal water may be limited by relatively low nutrient concentrations^26^, resulting in low abundance. In line with previous studies^27^, sediment hosted a higher abundance of microbes compared to waters, likely by providing rich nutrients and microhabitats.

Our sampling design allowed to assess the magnitude of community heterogeneity across distinct dimensions. The prokaryotic communities showed the highest variation among sampling regions (FR vs. TR vs. CR), modest variation among habitats (water vs. sediment), and the lowest variation between lifestyles (FL vs. PA). This conflicted with the findings of Griffin et al.^28^, who reported a higher degree of community similarity between upstream and downstream waters compared to between water and sediments/soils. The discrepancy could be attributed to the broader environmental gradients in our study area, particularly salinity, where spatial variation may overshadow habitat variation. Numerous studies have documented differences between FL and PA communities^29^, ^30^, ^31^, ^32^. Our results confirmed this in the Changjiang River. We further showed that the FL–PA community heterogeneity differed significantly along the river path, decreasing from the freshwater upstream to the lowest level at the transition region, before increasing again in the coastal region. This large fluctuation in community dissimilarities may indicate distinct frequencies of localized lifestyle exchange between FL and PA, rather than synchronous dispersal with river flow.

The distinct communities showed contrasting distribution patterns across spatial distances, as revealed by the distance–decay curves (Figure S6). While our sampling area extended to the coastal ocean, we anticipated the most significant community variation at the freshwater–seawater mixing zone (TR). This was indeed the case for FL and PA communities in water samples. However, although the prokaryotic community in sediments showed the fastest turnover rates, the most notable changes occurred over smaller spatial scales within FR, with no significant changes observed in TR. While previous studies have documented shifts in microbial community from freshwater to coastal seas, either in water or in sediments^33^, ^34^, our results indicated a significantly distinct spatial pattern between planktonic and benthic microbes. This difference is likely due to the effect of distinct disposition sources on the river sediment communities along the river flow and their varying sensitivity to change in environmental factors, such as salinity in this case.

### Distinct *β*‐diversity partitioning patterns between lifestyle/habitats

The richness effect was more important on FL compared to PA and benthic communities, and it remained at a high level along the river flow direction. This indicated that downstream microorganisms may be a subset of those from upstream that have undergone selection. Previous studies supported this notion, showing that freshwater communities can migrate to brackish estuaries during high river flows^25^, ^35^, where they were influenced by both seawater and terrestrial microorganisms. Thus, upstream microbes can act as seed banks for downstream microbial communities^36^.

The impact of taxonomic replacement on FL communities increased with geographic distance and even exceeded the richness effect in the coastal sea. This may be attributed to drastic environmental changes causing the loss of upstream taxa and the emergence of new taxa^37^. Compared to FL communities, taxonomic replacement had an overwhelming impact on PA and benthic microorganisms, likely due to their limited dispersal capacity, resulting in low sharing and species turnover between distantly related samples. This effect of taxonomic replacement further increased along the river flow, reflecting increased environmental niche partitioning similar to what has been observed in the FL communities.

### Co‐dominance of deterministic and stochastic processes in the prokaryotic community assembly

Deterministic and stochastic processes co‐dominated the prokaryotic community assembly in all regions and habitats, with the balance between these processes being affected by geographical and environmental scales^38^, ^39^. A large spatial scale and strong environmental gradients are more likely to result in the dominance of determinism^40^. The samples of our study covered a spatial range of ~1900 km, encompassing complex environmental dynamics along the river path and from freshwater to seawater that may reinforce deterministic processes. However, despite considerable environmental changes, it was notable that deterministic processes were predominantly manifested through the effect of homogeneous selection rather than heterogeneous selection. This may be explained by the river flow along the river path and the selective pressures exerted by homogeneous environmental conditions within the same sampling region.

With increasing spatial distance toward the coastal sea, there was a decrease in the influence of homogeneous selection but an increase in the influence of heterogeneous selection, which was expected due to the increasing salinity difference from freshwater to saltwater. The impact of heterogeneous selection showed only a subtle increase when salinity increased from 10 to 20 PSU, with a relatively evident increase observed under a salinity gradient of 0–35 PSU. This effect was more evident in the PA fraction than in the FL fraction, suggesting a high tolerance of freshwater microbes, especially FL microbes, to salinity changes. The exact salinity threshold that causes disruptive community turnover needs further elucidation by higher‐resolution salinity sampling. The increase of heterogeneous selection with increasing environmental gradients appeared to be more pronounced in sediments compared to waters. This observation is likely due to the complex sources received by sediments in transitional and coastal areas, and the dynamic depositional processes^41^ that may enhance environmental heterogeneity.

Dispersal limitation was dominant in stochastic processes, with its contribution increasing with spatial distance, consistent with the idea that dispersal is more difficult over larger distances^42^. The impact of dispersal limitation was more significant in benthic than planktonic communities, and in PA than FL communities, consistent with the weaker dispersal ability of microbes attached to particles and sediments^20^. Microbes adhered to particles face greater restrictions in movement into the surrounding environment^21^. Moreover, the spatial scale of the influence of dispersal limitation differed between PA and FL communities. Although FL microbes flow with water in the river, they experienced an increased effect of dispersal limitation with increasing spatial distance, while dispersal limitation can exert a higher effect over short spatial distances on PA microbes. This further underscored the low dispersal capacity of PA microbes and indicated a more complex assembly process for PA communities, which cannot be simplistically explained by spatial distance. It was important to note that the significant increase in spatial distance, where dispersal limitation became more pronounced, was concomitant with an escalation in salinity gradients. The difficulty in the establishment of microbial communities under salinity change may contribute to the effect of dispersal limitation generated by spatial segregation^4^.

### Potential interaction pattern revealed by co‐occurrence networks

Network complexity and connectivity have been found to be positively correlated with environmental heterogeneity^43^. We showed that prokaryotic communities in the coastal region had a more connected, complex, and coexisting network, likely reflecting a high degree of environmental heterogeneity due to the influence of freshwater discharge and convergence of various water masses. Under such heterogeneous environments, microbes tended to form more cooperative networks to share resources and transfer information^44^. This was in contrast to more homogeneous environments where microbes occupy similar biological ecological niches and result in fewer interactions but more competitive relationships^45^, ^46^. Supporting this, a previous study in reservoir sediments reported that microbial networks became more connected during the high‐flow seasons, likely due to increased environmental heterogeneity resulting from higher water flow^7^. In addition, we found differences in network structure between PA and FL communities. The higher connectivity and complexity of the PA network may be promoted by close physical contact and reflect a collaborative approach in the degradation of high‐molecular‐weight compounds. This is supported by the higher proportion of positive edges in the PA network indicative of more frequent synergistic interactions.

### Assessing the relative importance of biotic and abiotic factors

Environmental selection of microbial communities reflects a joint effect of abiotic factors and biotic interactions^2^. The latter is difficult to measure and is mostly neglected in explaining microbial community variations. In this study, we used the network topology of each sample as an index of biotic interaction and compared its explanatory power of community variations to abiotic factors via variance partitioning analysis (VPA) analysis. We found that addition of the biotic factors increased an explanation power of 18.36% and 18.98% on FL and PA community variations, respectively. Variance of the PA community explained by either pure biotic or pure abiotic factors was higher than that of the FL community. However, the variance of the FL community explained by the joint effect of these two factors was higher than that of the PA community. This indicated that abiotic and biotic factors had stronger interactions in shaping FL communities, whereas they tended to exert an individual effect on PA communities. A large part of community variance could not be explained despite addition of the biotic factors, indicating the possible role of other unmeasured environmental factors and interactions with other organisms such as eukaryotes. These unmeasured factors and their interactions may explain the disparities between the CCA and VPA results of the relative importance of environmental factors.

Microbial interactions may affect community assembly by maintaining or filtering microbial species^47^. Homogeneous selection showed a greater impact on FL taxa, as competition in more homogeneous environments can lead to the loss of rare species^47^, resulting in fewer observed species in FL communities. Although heterogeneous selection affects both FL and PA groups similarly, the complex microenvironments within particles may foster more close microbial interactions among PA microbes. In more heterogeneous environments, surviving species are more likely to promote aggregation and form more cooperative relationships within microbial communities^2^, which favors the maintenance of microbial diversity. Our results confirmed this, indicating that the network of PA microbes showed a greater proportion of positive and negative correlations compared to FL microbes. Individuals within the PA network demonstrated a stronger inclination toward collaboration and displayed higher diversity.

In summary, our study revealed the spatial dynamics of prokaryotic communities along a river–estuary–sea continuous, highlighting the significant influence of habitat and lifestyle types. We showed that spatial heterogeneity was more pronounced than variations caused by habitats (water vs. sediment) and lifestyles (FL vs. PA). The different communities showed contrasting spatial patterns; for example, the planktonic (both FL and PA) and benthic communities showed higher turnover in the freshwater–seawater mixing zone and the freshwater zone, respectively. These spatial dynamics were driven by different contributions of richness and the replacement effect, but were all primarily assembled by a dominant effect of homogeneous selection and dispersal limitation. At larger spatial distances, the influence of homogeneous selection decreased while the impact of dispersal limitation increased, with this trend being more pronounced in FL communities. This suggests that salinity barriers hinder the establishment of freshwater prokaryotic communities, thus contributing to dispersal limitation. In addition, while biotic and abiotic factors alone had a greater explanatory power for the PA community, their interactions had a greater explanatory power for the FL community. Overall, our findings provide new insights into the assembly of microbial communities in natural ecosystems characterized by high spatial connectivity and environmental heterogeneity.

## MATERIALS AND METHODS

### Study area, sampling, and environmental factors

A sampling campaign was conducted along the Changjiang River, China, from March 18 to April 14, 2021. This sampling was carried out in 51 stations along the Changjiang River basin (Zhu Tuo [ZT] to Nan Jing [NJ] stations), the Changjiang River estuary (Nan Tong [NT] to C9 stations), and its adjacent seas (Table S1). A portable global positioning system (GPS) was used to record the location of the sampling sites. For water sampling, only surface water was collected in the FR, and two layers, i.e., surface and bottom water, were collected in the TR and the CR. Water was successively filtered through a 3‐μm and a 0.22‐μm polycarbonate membrane (Millipore), representing PA and FL fractions^29^, ^48^, ^49^, respectively. After filtration, the membranes were transferred to cryogenic storage tubes and immediately placed in liquid nitrogen. Undisturbed surface sediments (0−2 cm) were collected by a box corer, carefully transferred into sterilized bags, and immediately stored at −20°C on board. After the cruise, the samples were transported to the laboratory and frozen at −80°C until DNA extraction. A total of 159 samples were collected, including 126 water and 33 sediment samples. Water depth (0−58 m), temperature (10.8–18.1°C), salinity (0−33.7 PSU), dissolved oxygen (8.36−273.57 μmol l^−1^), and chlorophyll *a* (0.36−8.43 μg l^−1^) were recorded using the AAQ171 multiparameter water quality sensor (JFE) (Table S1). Dissolved inorganic nutrients were analyzed using an AA3 autoanalyzer system.

### DNA extraction, PCR amplification, and Illumina MiSeq sequencing

DNA was extracted from filter membranes and sediments using the PowerSoil DNA Isolation Kit (Qiagen) and a FastPrep‐24 cell disrupter (MP Biomedicals) according to the manufacturers’ instructions. The quality, concentration, and purity of DNA were assessed by gel electrophoresis and a NanoDrop 2000 spectrophotometer (Thermo Scientific). The modified 515F (5′‐GTGYCAGCMGCCGCGGTAA‐3′) and 806R (5′‐ GGACTACNVGGGTWTCTAAT‐3′) primer pair^50^ was used to amplify the 16S rRNA gene V4 region. The PCR reaction system and conditions were according to the method described by Zhang et al.^37^. The PCR amplicons were purified using the AxyPrep DNA Gel Extraction Kit (Axygen Biosciences), combined in equimolar, and then sequenced on an Illumina MiSeq PE300 platform (Illumina), following the standard protocols provided by Majorbio Bio‐Pharm Technology Co. Ltd.

### Quantitative PCR (qPCR)

qPCR of the bacterial 16S rRNA gene was performed to detect the abundance of total bacteria in each sample using the primer set 967 F/1046R^51^. The total amount of each reaction well was 20 μl, which included 10 µl of SYBR Premix ExTaq II (2×), 0.4 µl of ROX Reference Dye II (50×) (TaKaRa), 0.5 µl of primers for each gene (10 µM), and 2 µl of template. The thermal cycling steps consisted of an initial denaturation at 94°C for 3 min, 35 cycles of 94°C for 30 s, 57°C for 45 s, and 72°C for 30 s, and a final extension at 72°C for 5 min. All assays were performed using the QuantStudioTM 5 Real‐Time PCR System (Applied Biosystems, USA) with three parallels per sample and negative controls. Standard curves were generated with ten‐fold serial dilutions of a linear plasmid containing a single copy of the bacterial 16SrRNA gene; the curves showed good linear relationships (*R* ^2^ > 0.99) and amplification efficiencies in the range of 90%−105%.

### Sequence processing

The raw reads were quality‐filtered and merged using fastp v0.19.6^52^ and FLASH v1.2.7^53^, respectively, based on the following criteria: (i) reads were truncated at positions where the average quality score dropped below 20 within a sliding window of 50 bp; (ii) sequences with overlap lengths greater than 10 bp were merged, allowing a maximum mismatch ratio of 0.2; and (iii) samples were assigned based on barcodes (exact matches) and primers (up to two nucleotide mismatches). The high‐quality sequences were then processed to generate ASVs using the default DADA2 workflow on the Qiime2 platform^54^. Taxonomic classification for each ASV representative sequence was performed with classify‐sklearn^55^ using the Silva 16S rRNA database^56^ (v138, confidence threshold of 0.7) and ASVs annotated as mitochondria or chloroplasts were removed. ASVs with fewer than 10 sequences across all samples were discarded. Sequencing of the 16S rRNA gene yielded 9,532,832 reads across all samples, which were clustered into 31,439 ASVs after rarefying to 13,089 reads per sample. The rarefaction curves for each sample were all near the horizontal position (Figure S9), indicating that the sequencing depth was sufficient to accurately represent the intact prokaryotic communities.

### Prokaryotic community composition and diversity analysis

Based on the standardized ASV matrices, alpha diversity indices, including Shannon, observed ASVs, PD and Pielou's evenness, were calculated using the “vegan” package in R software v4.1.2. Significant differences were tested using Tukey's HSD test. The top 1000 most abundant ASVs in each habitat were selected and their specificity and occupancy were calculated^14^, ^57^. Specificity refers to the average abundance of a species (S) within a habitat sample (H, such as each region or lifestyle sample), while occupancy is the proportion of samples within habitat H that contain species S. The specific algorithm is as follows: Specificity is calculated as the ratio of the average ASV count of species S in all samples of habitat H to the sum of average count of species S across all habitats. Occupancy is determined as the ratio of the number of samples in habitat H that contain species S to the total number of samples in habitat H. We used 70% and 30% as cutoffs for categorizing high and low specificity and occupancy, respectively.

For community comparison, NMDS was conducted using Bray–Curtis distances using the R package “vegan.” PERMANOVA was used to evaluate the significant differences between groups. Total *β*‐diversity and its components, replacement (*β*_repl_) and richness (*β*_rich_), were calculated based on the Jaccard distance using the R package “adespatial”^58^. We defined horizontal *β*‐diversity as *β*‐diversity between the sum of ASVs in the most upstream site (ZT) and each further site.

Pairwise geographic distances were computed from the latitude and longitude coordinates of sampling points using the “geosphere” package in R. The “ggplot2” package was used to visualize the relationship between community similarities based on the Bray–Curtis distance and geographic distances, and the correlation between the two variables was calculated by the Spearman correlation coefficient.

CCA was performed to evaluate the linkages between planktonic community structure and environmental parameters using the “vegan” package in R. VPA was conducted to compare the effects of environmental and biological factors on community structures. As covariation might occur among these environmental factors, the variance inflation factor (VIF) was used for selecting explanatory variables to minimize the influence of collinearity. Collinear environmental factors were sequentially eliminated through VIF analysis until all the remaining explanatory variables had VIF values < 10^20^. Wherever possible, *p* < 0.05 (adjusted by the false discovery rate) was considered to indicate significant correlations. The correlation between environmental factors and Mantel tests between environmental factors and microbial communities based on Bray–Curtis were performed using the “ggcor 0.9.8” package.

### Null model

The null modeling framework based on iCAMP was performed to quantify the impact of the assembly process on microbial community structure. The obtained high‐quality sequences were subjected to sequence alignment and a maximum likelihood tree was constructed using the FastTree algorithm. Based on the modified stochastic processes calculated by pNST, we set the bin size limit as 64. Using the “iCAMP” package in R, the beta Net Relatedness Index (βNRI) was calculated. The relative importance of each ecological process was assessed by βNRI and Raup–Crick (RC_bray_) values based on the Bray distance. Values of βNRI > + 1.96 or < −1.96 indicate that deterministic processes influence the community structure, whereas values between −1.96 and +1.96 indicate that stochastic processes are mainly influential^59^. The relative importance of the stochastic process was further assessed by combining βNRI with Raup–Crick (RC_bray_). |βNRI| values ≤ 1.96 with RC_bray_ < −0.95 indicate that the turnover of community composition is dominated by homogenizing dispersal, −0.95 ≤ RC_bray_ ≤ 0.95 indicates that ecological drift dominates the assembly of the community, and RC_bray_ > 0.95 represents dispersal limitation^59^.

### Co‐occurrence network analysis

Co‐occurrence networks were constructed using the “igraph” and “Hmisc” packages in R, based on Spearman's correlation matrices of water samples, and the networks were visualized by Gephi (v0.9.4 beta, http://gephi.org). To reduce the complexity of the network, only ASVs with a relative abundance greater than 0.01% and a frequency of occurrence greater than 20% of samples were retained. The appropriate similarity thresholds of 0.76 and 0.86 for the entire and regional networks of FL/PA, respectively, were identified using random matrix theory (RMT)^60^. The network‐level (average degree, clustering coefficient, average path length, modularity, graph density, network diameter, betweenness centralization, and degree centralization) topological features were calculated. In addition, the subnetwork for each sample was extracted using the induced_subgraph function in the “igraph” package, and then the topological features of each sub‐net were computed for the following 11 parameters: average degree, average path length, betweenness centrality and closeness centrality, clustering coefficient, modularity, degree centralization, and diameter. The above characteristic parameters, serving as interaction indices, were analyzed by VPA together with environmental factors to analyze the relative importance of biotic and environmental factors in community variations. In addition, network robustness was analyzed by simulation of species extinction through random removal of 50% of nodes as described by Yuan et al.^61^.

## AUTHOR CONTRIBUTIONS

**Jiao Liu:** Writing—original draft; visualization; formal analysis; investigation. **Peng Yao:** Investigation; writing—review and editing; resources. **Jinmei Liu:** Visualization; formal analysis. **Gaoyang Ren:** Investigation; formal analysis; visualization. **Xiao‐Hua Zhang:** Conceptualization; writing—review and editing; resources. **Jiwen Liu:** Conceptualization; funding acquisition; writing—review and editing; project administration; supervision; resources.

## ETHICS STATEMENT

No animal or human subjects were involved in this study.

## CONFLICT OF INTERESTS

The authors declare no conflict of interests.

## Supporting information

mlife‐2024‐0160.

## Data Availability

The data that support the findings of this study have been submitted to the National Omics Data Encyclopedia (https://www.biosino.org/node) under accession number OEP005174.

## References

[1] Louca S , Polz MF , Mazel F , Albright MBN , Huber JA , O'Connor MI , et al. Function and functional redundancy in microbial systems. Nat Ecol Evol. 2018;2:936–943. 29662222 10.1038/s41559-018-0519-1

[2] Nemergut DR , Schmidt SK , Fukami T , O'Neill SP , Bilinski TM , Stanish LF , et al. Patterns and processes of microbial community assembly. Microbiol Mol Biol Rev. 2013;77:342–356. 24006468 10.1128/MMBR.00051-12 PMC3811611

[3] Stegen JC , Lin X , Fredrickson JK , Chen X , Kennedy DW , Murray CJ , et al. Quantifying community assembly processes and identifying features that impose them. ISME J. 2013;7:2069–2079. 23739053 10.1038/ismej.2013.93 PMC3806266

[4] Zhou J , Ning D . Stochastic community assembly: does it matter In microbial ecology? Microbiol Mol Biol Rev. 2017;81:4. 10.1128/MMBR.00002-17 PMC5706748 29021219

[5] Burns AR , Stephens WZ , Stagaman K , Wong S , Rawls JF , Guillemin K , et al. Contribution of neutral processes to the assembly of gut microbial communities in the zebrafish over host development. ISME J. 2016;10:655–664. 26296066 10.1038/ismej.2015.142 PMC4817674

[6] Liu J , Meng Z , Liu X , Zhang XH . Microbial assembly, interaction, functioning, activity and diversification: a review derived from community compositional data. Mar Life Sci Technol. 2019;1:112–128.

[7] Li Y , Gao Y , Zhang W , Wang C , Wang P , Niu L , et al. Homogeneous selection dominates the microbial community assembly in the sediment of the Three Gorges Reservoir. Sci Total Environ. 2019;690:50–60. 31284194 10.1016/j.scitotenv.2019.07.014

[8] Yang Y . Emerging patterns of microbial functional traits. Trends Microbiol. 2021;29:874–882. 34030967 10.1016/j.tim.2021.04.004

[9] Tilman D . Niche tradeoffs, neutrality, and community structure: a stochastic theory of resource competition, invasion, and community assembly. Proc Natl Acad Sci USA. 2004;101:10854–10861. 15243158 10.1073/pnas.0403458101 PMC503710

[10] Zhang W , Li Y , Wang C , Wang P , Hou J , Yu Z , et al. Modeling the biodegradation of bacterial community assembly linked antibiotics in river sediment using a deterministic stochastic combined model. Environ Sci Technol. 2016;50:8788–8798. 27428250 10.1021/acs.est.6b01573

[11] Stadler M , del Giorgio PA . Terrestrial connectivity, upstream aquatic history and seasonality shape bacterial community assembly within a large boreal aquatic network. ISME J. 2022;16:937–947. 34725445 10.1038/s41396-021-01146-y PMC8941091

[12] Gao Y , Zhang W , Li Y , Wu H , Yang N , Hui C . Dams shift microbial community assembly and imprint nitrogen transformation along the Yangtze River. Water Res. 2021;189:116579. 33160238 10.1016/j.watres.2020.116579

[13] Zhang S , Li K , Hu J , Wang F , Chen D , Zhang Z , et al. Distinct assembly mechanisms of microbial sub‐communities with different rarity along the Nu River. J Soils Sediments. 2022;22:1530–1545.

[14] Gweon HS , Bowes MJ , Moorhouse HL , Oliver AE , Bailey MJ , Acreman MC , et al. Contrasting community assembly processes structure lotic bacteria metacommunities along the river continuum. Environ Microbiol. 2021;23:484–498. 33258525 10.1111/1462-2920.15337 PMC7898806

[15] Fortunato CS , Crump BC . Microbial gene abundance and expression patterns across a river to ocean salinity gradient. PLoS One. 2015;10:e0140578. 26536246 10.1371/journal.pone.0140578 PMC4633275

[16] Tee HS , Waite D , Lear G , Handley KM . Microbial river‐to‐sea continuum: gradients in benthic and planktonic diversity, osmoregulation and nutrient cycling. Microbiome. 2021;9:190. 34544488 10.1186/s40168-021-01145-3 PMC8454136

[17] Cloern JE , Jassby AD , Schraga TS , Nejad E , Martin C . Ecosystem variability along the estuarine salinity gradient: examples from long‐term study of San Francisco Bay. Limnol Oceanogr. 2017;62:S272–S291.

[18] Zinger L , Amaral‐Zettler LA , Fuhrman JA , Horner‐Devine MC , Huse SM , Welch DBM , et al. Global patterns of bacterial beta‐diversity in seafloor and seawater ecosystems. PLoS One. 2011;6:e24570. 21931760 10.1371/journal.pone.0024570 PMC3169623

[19] Zinger L , Boetius A , Ramette A . Bacterial taxa‐area and distance‐decay relationships in marine environments. Mol Ecol. 2014;23:954–964. 24460915 10.1111/mec.12640 PMC4230465

[20] Liu J , Zhu S , Liu X , Yao P , Ge T , Zhang XH . Spatiotemporal dynamics of the archaeal community in coastal sediments: assembly process and co‐occurrence relationship. ISME J. 2020;14:1463–1478. 32132664 10.1038/s41396-020-0621-7 PMC7242467

[21] Liu J , Wang X , Liu J , Liu X , Zhang XH , Liu J . Comparison of assembly process and co‐occurrence pattern between planktonic and benthic microbial communities in the Bohai Sea. Front Microbiol. 2022;13:1003623. 36386657 10.3389/fmicb.2022.1003623 PMC9641972

[22] Mansour I , Heppell CM , Ryo M , Rillig MC . Application of the microbial community coalescence concept to riverine networks. Biol Rev. 2018;93:1832–1845. 29700966 10.1111/brv.12422

[23] Besemer K , Singer G , Quince C , Bertuzzo E , Sloan W , Battin TJ . Headwaters are critical reservoirs of microbial diversity for fluvial networks. Proc R Soc B Biol Sci. 2013;280:20131760. 10.1098/rspb.2013.1760 PMC3790480 24089333

[24] Wang S , Hou W , Jiang H , Huang L , Dong H , Chen S , et al. Microbial diversity accumulates in a downstream direction in the Three Gorges Reservoir. J Environ Sci. 2021;101:156–167. 10.1016/j.jes.2020.08.006 33334511

[25] Mason OU , Canter EJ , Gillies LE , Paisie TK , Roberts BJ . Mississippi River plume enriches microbial diversity in the northern Gulf of Mexico. Front Microbiol. 2016;7:1048. 27458442 10.3389/fmicb.2016.01048 PMC4936242

[26] Zhou X , Ge J , Wallhead P , Shi S . Changes in nutrient concentration resulting from floods and their impact on the Estuary‐Sea Continuum. J Geophys Res Oceans. 2023;128:e2022JC019327.

[27] Miettinen H , Bomberg M , Nyyssönen M , Reunamo A , Jørgensen KS , Vikman M . Oil degradation potential of microbial communities in water and sediment of Baltic Sea coastal area. PLoS One. 2019;14:e0218834. 31265451 10.1371/journal.pone.0218834 PMC6605675

[28] Griffin JS , Lu N , Sangwan N , Li A , Dsouza M , Stumpf AJ , et al. Microbial diversity in an intensively managed landscape is structured by landscape connectivity. FEMS Microbiol Ecol. 2017;93:fix120. 10.1093/femsec/fix120 28961974

[29] Wang Y , Pan J , Yang J , Zhou Z , Pan Y , Li M . Patterns and processes of free‐living and particle‐associated bacterioplankton and archaeaplankton communities in a subtropical river‐bay system in South China. Limnol Oceanogr. 2020;65:S161–S179.

[30] Jain A , Balmonte JP , Singh R , Bhaskar PV , Krishnan KP . Spatially resolved assembly, connectivity and structure of particle‐associated and free‐living bacterial communities in a high Arctic fjord. FEMS Microbiol Ecol. 2021;97:fiab139. 34626180 10.1093/femsec/fiab139 PMC8536490

[31] Roth Rosenberg D , Haber M , Goldford J , Lalzar M , Aharonovich D , Al‐Ashhab A , et al. Particle‐associated and free‐living bacterial communities in an oligotrophic sea are affected by different environmental factors. Environ Microbiol. 2021;23:4295–4308. 34036706 10.1111/1462-2920.15611

[32] Liu J , Huang F , Liu J , Liu X , Lin R , Zhong X , et al. Phylotype resolved spatial variation and association patterns of planktonic Thaumarchaeota in eastern Chinese marginal seas. Mar Life Sci Technol. 2023;5:257–270. 37275536 10.1007/s42995-023-00169-y PMC10232715

[33] Figueroa D , Capo E , Lindh MV , Rowe OF , Paczkowska J , Pinhassi J , et al. Terrestrial dissolved organic matter inflow drives temporal dynamics of the bacterial community of a subarctic estuary (northern Baltic Sea). Environ Microbiol. 2021;23:4200–4213. 33998121 10.1111/1462-2920.15597

[34] Blais M‐A , Vincent WF , Vigneron A , Labarre A , Matveev A , Coelho LF , et al. Diverse winter communities and biogeochemical cycling potential in the under‐ice microbial plankton of a subarctic river‐to‐sea continuum. Microbiol Spectr. 2024;12:e04160‐04123. 38511950 10.1128/spectrum.04160-23 PMC11210273

[35] Crump BC , Hopkinson CS , Sogin ML , Hobbie JE . Microbial biogeography along an estuarine salinity gradient: combined influences of bacterial growth and residence time. Appl Environ Microbiol. 2004;70:1494–1505. 15006771 10.1128/AEM.70.3.1494-1505.2004 PMC365029

[36] de Oliveira LFV , Margis R . The source of the river as a nursery for microbial diversity. PLoS One. 2015;10:e0120608. 25803426 10.1371/journal.pone.0120608 PMC4372583

[37] Zhang Y , Yao P , Sun C , Li S , Shi X , Zhang XH , et al. Vertical diversity and association pattern of total, abundant and rare microbial communities in deep‐sea sediments. Mol Ecol. 2021;30:2800–2816. 33960545 10.1111/mec.15937 PMC8251536

[38] Hanson CA , Fuhrman JA , Horner‐Devine MC , Martiny JBH . Beyond biogeographic patterns: processes shaping the microbial landscape. Nat Rev Microbiol. 2012;10:497–506. 22580365 10.1038/nrmicro2795

[39] Wan W , Gadd GM , Gu J‐D , Liu W , Chen P , Zhang Q , et al. Beyond biogeographic patterns: processes shaping the microbial landscape in soils and sediments along the Yangtze River. mLife. 2023;2:89–100. 38818339 10.1002/mlf2.12062 PMC10989888

[40] Lu M , Wang X , Li H , Jiao JJ , Luo X , Luo M , et al. Microbial community assembly and co‐occurrence relationship in sediments of the river‐dominated estuary and the adjacent shelf in the wet season. Environ Pollut. 2022;308:119572. 35661808 10.1016/j.envpol.2022.119572

[41] Zhao B , Yao P , Bianchi TS , Xu Y , Liu H , Mi T , et al. Early diagenesis and authigenic mineral formation in mobile muds of the Changjiang Estuary and adjacent shelf. J Mar Syst. 2017;172:64–74.

[42] Heino J , Melo AS , Siqueira T , Soininen J , Valanko S , Bini LM . Metacommunity organisation, spatial extent and dispersal in aquatic systems: patterns, processes and prospects. Freshwater Biol. 2015;60:845–869.

[43] Mougi A , Kondoh M . Diversity of interaction types and ecological community stability. Science. 2012;337:349–351. 22822151 10.1126/science.1220529

[44] Morriën E , Hannula SE , Snoek LB , Helmsing NR , Zweers H , de Hollander M , et al. Soil networks become more connected and take up more carbon as nature restoration progresses. Nat Commun. 2017;8:14349. 28176768 10.1038/ncomms14349 PMC5309817

[45] Cherif M , Loreau M . Stoichiometric constraints on resource use, competitive interactions, and elemental cycling in microbial decomposers. Am Nat. 2007;169:709–724. 17479458 10.1086/516844

[46] Freilich S , Zarecki R , Eilam O , Segal ES , Henry CS , Kupiec M , et al. Competitive and cooperative metabolic interactions in bacterial communities. Nat Commun. 2011;2:589. 22158444 10.1038/ncomms1597

[47] Petro C , Starnawski P , Schramm A , Kjeldsen K . Microbial community assembly in marine sediments. Aquat Microb Ecol. 2017;79:177–195.

[48] D'ambrosio L , Ziervogel K , MacGregor B , Teske A , Arnosti C . Composition and enzymatic function of particle‐associated and free‐living bacteria: a coastal/offshore comparison. ISME J. 2014;8:2167–2179. 24763371 10.1038/ismej.2014.67 PMC4992077

[49] Geller‐McGrath D , Mara P , Taylor GT , Suter E , Edgcomb V , Pachiadaki M . Diverse secondary metabolites are expressed in particle‐associated and free‐living microorganisms of the permanently anoxic Cariaco Basin. Nat Commun. 2023;14:656. 36746960 10.1038/s41467-023-36026-w PMC9902471

[50] Walters W , Hyde ER , Berg‐Lyons D , Ackermann G , Humphrey G , Parada A , et al. Improved bacterial 16S rRNA gene (V4 and V4‐5) and fungal internal transcribed spacer marker gene primers for microbial community surveys. mSystems. 2016;1:e00009‐15. 10.1128/mSystems.00009-15 PMC5069754 27822518

[51] Sogin ML , Morrison HG , Huber JA , Welch DM , Huse SM , Neal PR , et al. Microbial diversity in the deep sea and the underexplored “rare biosphere. Proc Natl Acad Sci USA. 2006;103:12115–12120. 16880384 10.1073/pnas.0605127103 PMC1524930

[52] Chen S , Zhou Y , Chen Y , Gu J . fastp: an ultra‐fast all‐In‐one FASTQ preprocessor. Bioinformatics. 2018;34:i884–i890. 30423086 10.1093/bioinformatics/bty560 PMC6129281

[53] Magoč T , Salzberg SL . FLASH: fast length adjustment of short reads to improve genome assemblies. Bioinformatics. 2011;27:2957–2963. 21903629 10.1093/bioinformatics/btr507 PMC3198573

[54] Bolyen E , Rideout JR , Dillon MR , Bokulich NA , Abnet CC , Al‐Ghalith GA , et al. Reproducible, interactive, scalable and extensible microbiome data science using QIIME 2. Nat Biotechnol. 2019;37:852–857. 31341288 10.1038/s41587-019-0209-9 PMC7015180

[55] Bokulich NA , Kaehler BD , Rideout JR , Dillon M , Bolyen E , Knight R , et al. Optimizing taxonomic classification of marker‐gene amplicon sequences with QIIME 2′s q2‐feature‐classifier plugin. Microbiome. 2018;6:90. 29773078 10.1186/s40168-018-0470-z PMC5956843

[56] Quast C , Pruesse E , Yilmaz P , Gerken J , Schweer T , Yarza P , et al. The SILVA ribosomal RNA gene database project: improved data processing and web‐based tools. Nucleic Acids Res. 2012;41:D590–D596. 23193283 10.1093/nar/gks1219 PMC3531112

[57] Dufrene M , Legendre P . Species assemblages and indicator species: the need for a flexible asymmetrical approach. Ecol Monograph. 1997;67:345–366.

[58] Shen C , Gunina A , Luo Y , Wang J , He JZ , Kuzyakov Y , et al. Contrasting patterns and drivers of soil bacterial and fungal diversity across a mountain gradient. Environ Microbiol. 2020;22:3287–3301. 32436332 10.1111/1462-2920.15090

[59] Ning D , Yuan M , Wu L , Zhang Y , Guo X , Zhou X , et al. A quantitative framework reveals ecological drivers of grassland microbial community assembly in response to warming. Nat Commun. 2020;11:4717. 32948774 10.1038/s41467-020-18560-z PMC7501310

[60] Zhou JZ , Deng Y , Luo F , He ZL , Yang YF . Phylogenetic molecular ecological network of soil microbial communities in response to elevated CO_2_ . mBio. 2011;2:10–1128. 10.1128/mBio.00122-11 PMC3143843 21791581

[61] Yuan MM , Guo X , Wu L , Zhang Y , Xiao N , Ning D , et al. Climate warming enhances microbial network complexity and stability. Nat Clim Change. 2021;11:343–348.

