# Supplementary material for: Habitat and lifestyle affect the spatial dynamics of prokaryotic communities along a river–estuary–sea continuum
Source: mLife. 2025 Jun 20;4(3):305–18. doi: 10.1002/mlf2.70017 (PMC12207907; doi:10.1002/mlf2.70017)
Supplement: Supplementary file 1 — mlife‐2024‐0160. [file MLF2-4-305-s001.pdf]

# Supplementary

## Habitat and lifestyle affect the spatial dynamics of prokaryotic communities along a river-estuary-sea continuum

Jiao Liu[1], Peng Yao[2,3], Jinmei Liu[1], Gaoyang Ren[1], Xiao-Hua Zhang[1,2,4], Jiwen Liu[1,2,4]*

**Figure S1** Alpha diversity indices of prokaryotic communities in different regions (freshwater region [FR] *vs* transition region [TR] *vs* coastal region [CR]) and different lifestyles (free-living [FL] *vs* particle-associated [PA]).
**Figure S2** Compositions of microbial communities inhabiting water and sediment. (A) Relative abundance of the top 12 microbial phyla. (B) Differential distribution of microbial phyla between water and sediment across all samples.
**Figure S3** Relative abundance of microbial phyla between FL and PA communities.
**Figure S4** Prevalence and specificity of ASV distributions. (A) The SPEC-OCCU plots showing the 1000 most abundant ASVs in communities of different habitats/lifestyles. (B) Relative abundance and taxonomic classification of specialist ASVs in different lifestyles.
**Figure S5** Community dissimilarities between FL and PA communities along the river flow. The light pink and dark pink represent samples from the surface water and bottom water, respectively.
**Figure S6** Distance-decay relationship of microbial communities in different regions based on Bray-Curtis dissimilarity.
**Figure S7** $\beta$-Diversity partitioning pattern for the top five most abundant phyla.
**Figure S8** Robustness measured as the proportion of taxa remained with 50% of the taxa randomly removed from each of the empirical MENs.
**Figure S9** The rarefaction curve of microbial sequences.

For Review Only

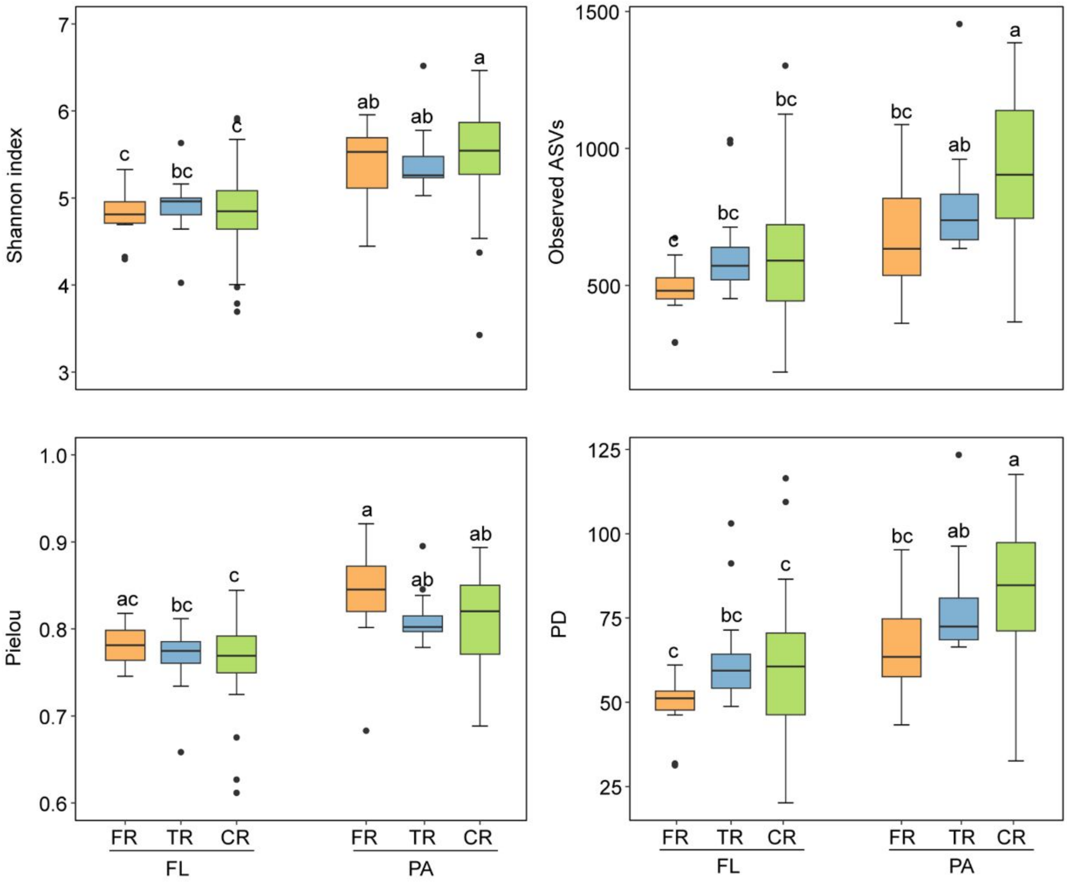


**Figure S1** Alpha diversity indices of prokaryotic communities in different regions (freshwater region [FR] *vs* transition region [TR] *vs* coastal region [CR]) and different lifestyles (free-living [FL] *vs* particle-associated [PA]).

For Review Only

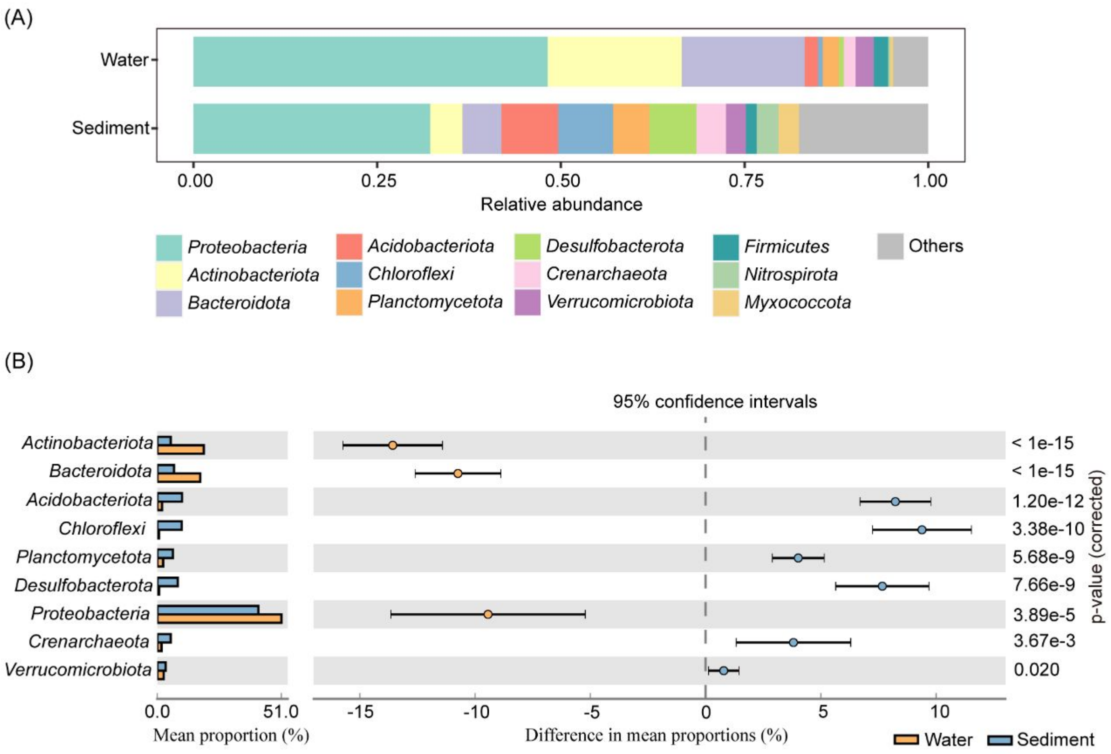


**Figure S2** Compositions of microbial communities inhabiting water and sediment. (A) Relative abundance of the top 12 microbial phyla. (B) Differential distribution of microbial phyla between water and sediment across all samples.

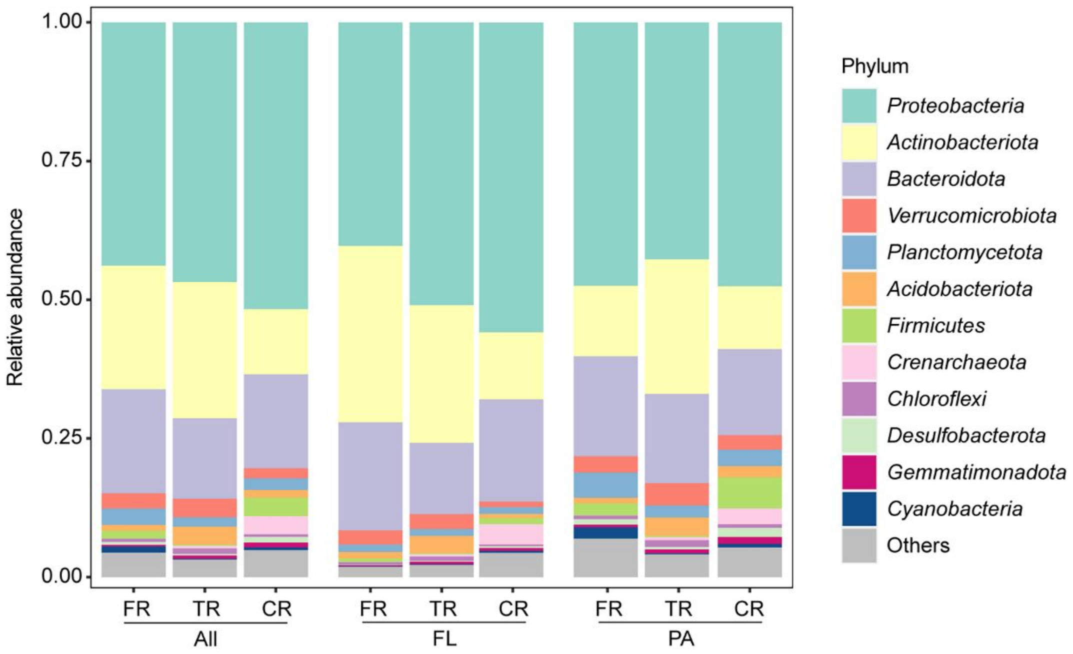


**Figure S3** Relative abundance of microbial phyla between FL and PA communities.

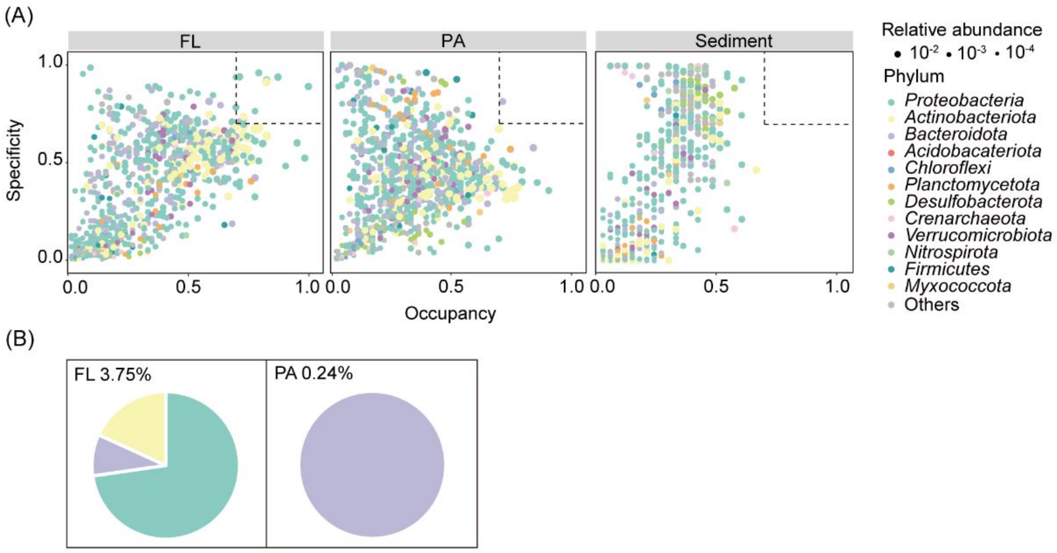


**Figure S4** Prevalence and specificity of ASV distributions. (A) The SPEC-OCCU plots showing the 1000 most abundant ASVs in communities of different habitats/lifestyles. (B) Relative abundance and taxonomic classification of specialist ASVs in different lifestyles.

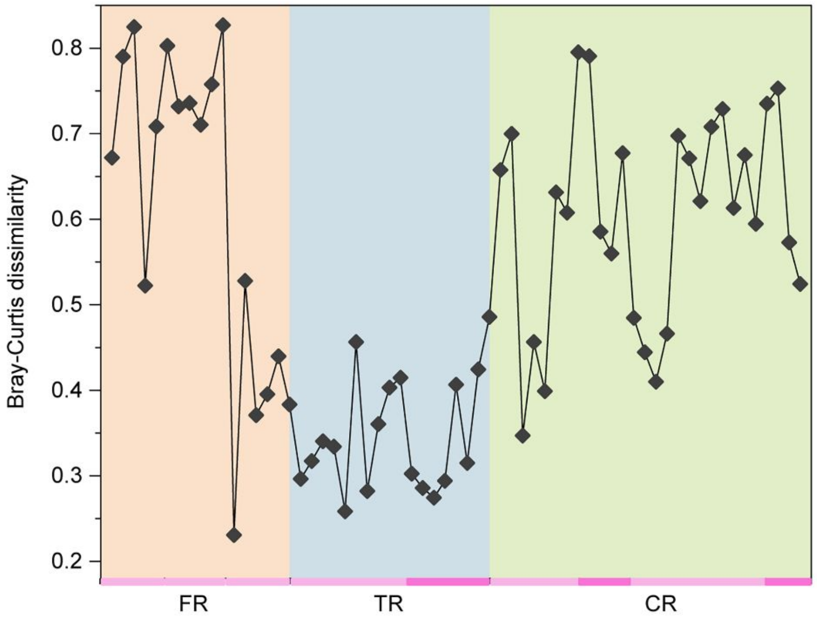


**Figure S5** Community dissimilarities between FL and PA communities along the river flow. The light pink and dark pink represent samples from the surface water and bottom water, respectively.

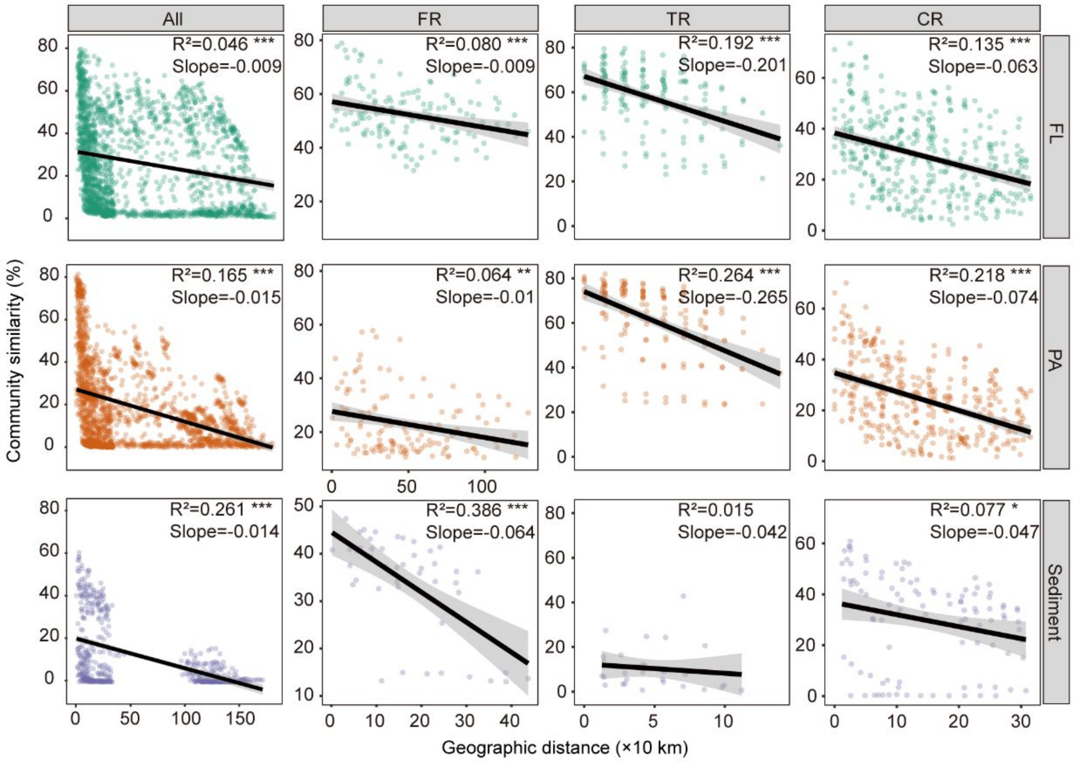


**Figure S6** Distance-decay relationship of microbial communities in different regions based on Bray-Curtis dissimilarity.

view Only

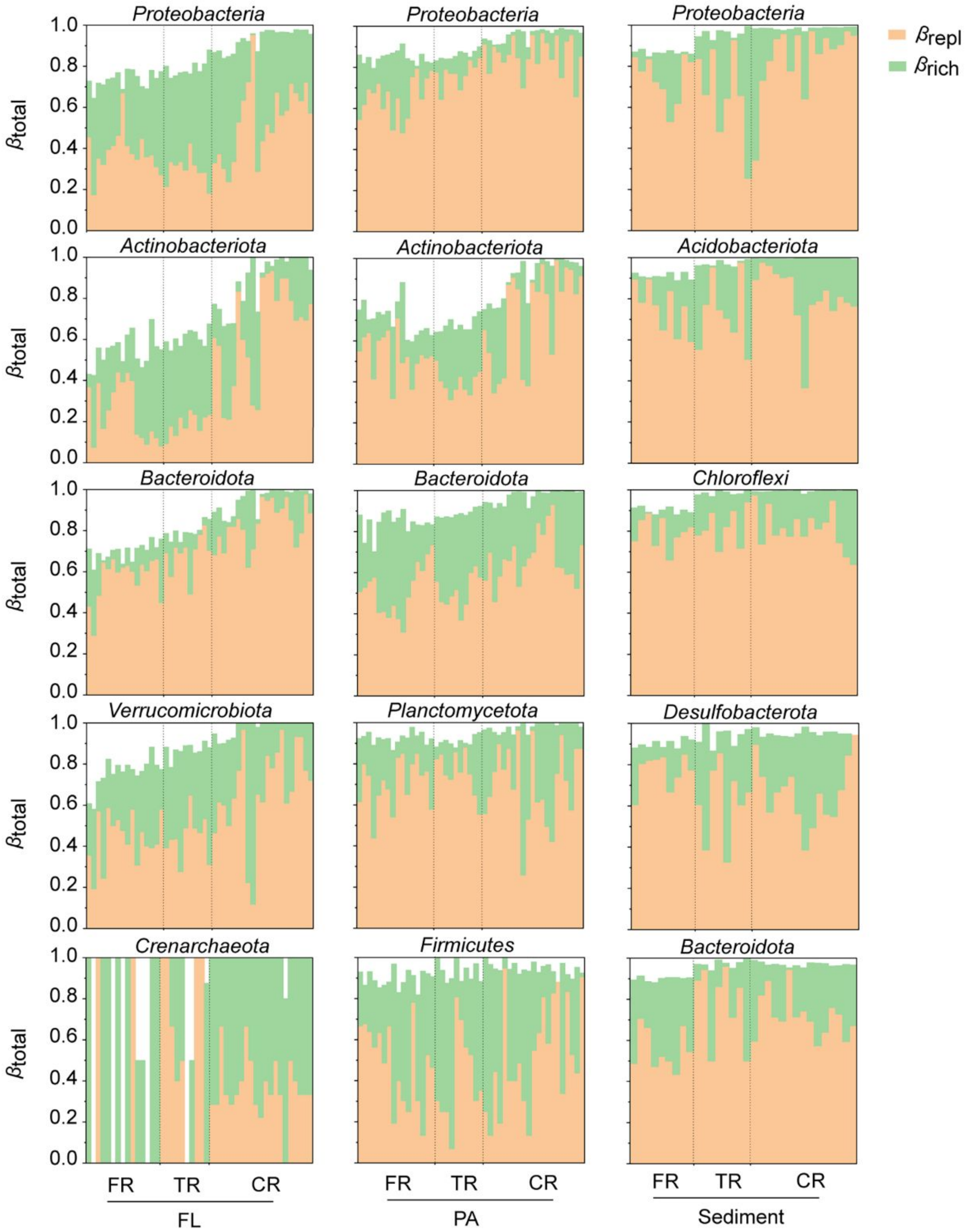


**Figure S7** *β*-Diversity partitioning pattern for the top five most abundant phyla.

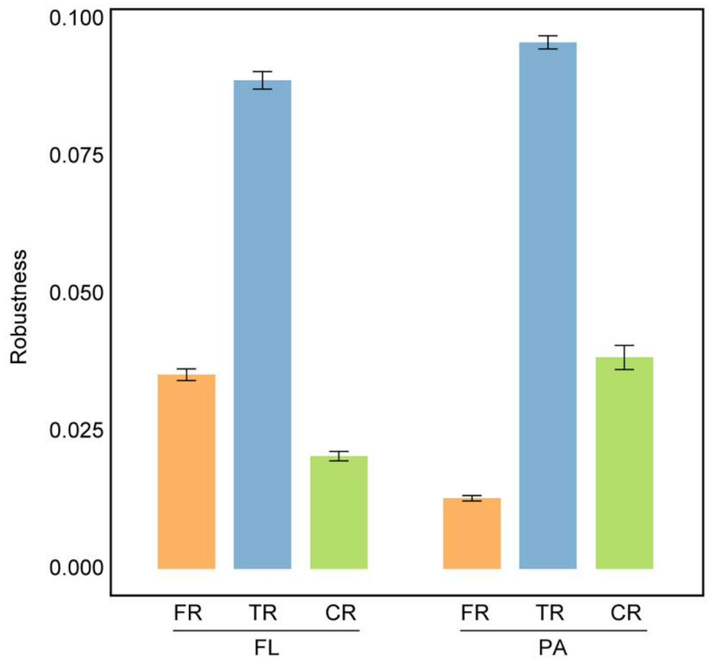


**Figure S8** Robustness measured as the proportion of taxa remained when 50% of the taxa were randomly removed from each of the empirical network.

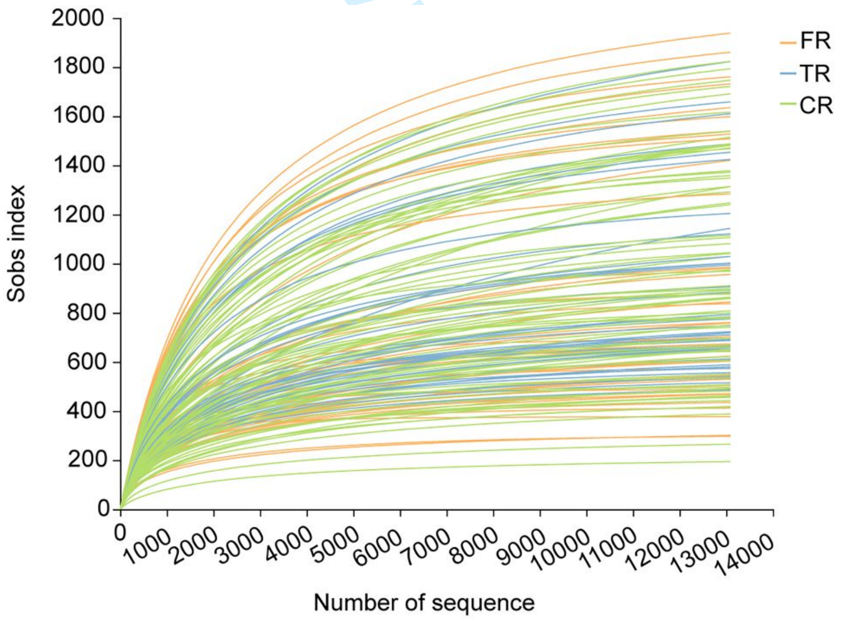


**Figure S9** The rarefaction curve of microbial sequences.

For Review Only

**Table S1** Sampling location and environmental parameters for each site.

| Station | Longitude (°E) | Latitude (°N) | Depth (m) | Surface sediment | Sal | Temp (°C) | pH | DO (µmol/L) | Chl a (µg/L) | Nutrients (µmol/L) | | | | |
|---|---|---|---|---|---|---|---|---|---|---|---|---|---|---|
| | | | | | | | | | | $NH_4^+$ | $NO_3^-$ | $SiO_3^{2-}$ | $NO_2^-$ | $PO_4^{3-}$ |
| ZT | 105.857 | 29.014 | 0 | — | 0.00 | 15.00 | 8.13 | 218.35 | 2.21 | 5.77 | 89.55 | 95.79 | 1.22 | 0.60 |
| BB | 106.447 | 29.826 | 0 | — | 0.00 | 15.00 | 8.44 | 273.57 | 3.67 | 1.13 | 77.39 | 80.72 | 1.17 | 0.09 |
| XK | 106.616 | 29.571 | 0 | 0-2 cm | 0.00 | 16.86 | 8.22 | 210.89 | 2.80 | 4.75 | 89.57 | 91.16 | 0.81 | 0.49 |
| WL | 107.824 | 29.295 | 0 | — | 0.00 | 13.97 | 8.06 | 204.91 | 0.84 | 3.65 | 152.93 | 81.19 | 0.87 | 0.93 |
| FD | 107.730 | 29.879 | 0 | 0-2 cm | 0.00 | 15.58 | 8.12 | 210.23 | 0.66 | 5.67 | 116.69 | 86.53 | 0.98 | 0.71 |
| ZX | 108.053 | 30.298 | — | 0-2 cm | — | — | — | — | — | — | — | — | — | — |
| WZ | 108.417 | 30.722 | 0 | 0-2 cm | 0.00 | 15.02 | 8.22 | 205.93 | 2.32 | 3.87 | 115 | 82.6 | 1.07 | 0.60 |
| YY | 108.639 | 30.949 | — | 0-2 cm | — | — | — | — | — | — | — | — | — | — |
| FJ | 109.409 | 30.986 | 0 | 0-2 cm | 0.00 | 14.65 | 8.25 | 201.16 | 1.64 | 2.83 | 114.29 | 90.5 | 2.24 | 0.76 |
| DNH | 109.893 | 31.120 | — | 0-2 cm | — | — | — | — | — | — | — | — | — | — |
| BD | 110.324 | 31.039 | 0 | 0-2 cm | 0.00 | 14.00 | 8.16 | 197.12 | 0.69 | 0.48 | 111.93 | 99.38 | 2.74 | 0.63 |
| XX | 110.328 | 31.057 | 0 | 0-2 cm | 0.00 | 15.52 | 8.52 | 227.49 | 1.09 | 9.74 | 97.88 | 86.38 | 0.69 | 0.58 |
| ZG | 110.968 | 30.863 | 0 | 0-2 cm | 0.00 | 12.93 | 8.22 | 203.90 | 0.61 | 1.77 | 115.36 | 102.26 | 0.17 | 0.75 |
| YC | 111.284 | 30.690 | 0 | — | 0.00 | 13.39 | 8.19 | 208.14 | 1.41 | 1.57 | 115.12 | 102.67 | 0.18 | 0.82 |
| YY | 113.228 | 29.537 | 0 | — | 0.00 | 14.92 | 8.09 | 203.59 | 2.39 | 11.84 | 102.87 | 97.87 | 2.81 | 0.51 |
| WH | 114.326 | 30.614 | 0 | — | 0.00 | 15.58 | 8.05 | 205.57 | 2.29 | 6.36 | 103.08 | 104.16 | 1.81 | 0.69 |
| JJ | 115.980 | 29.725 | 0 | — | 0.00 | 14.98 | 8.19 | 201.38 | 2.49 | 8.83 | 107.15 | 97.20 | 1.72 | 0.70 |
| HK | 116.216 | 29.739 | 0 | — | 0.00 | 18.19 | 7.98 | 191.05 | 8.43 | 9.41 | 86.96 | 124.51 | 2.51 | 0.73 |
| DT | 117.626 | 30.767 | 0 | — | 0.00 | 15.00 | 8.07 | 200.26 | 1.85 | 8.98 | 105.12 | 100.63 | 2.21 | 0.74 |
| NJ | 118.740 | 32.109 | 0 | — | 0.00 | 15.50 | 8.03 | 191.84 | 1.10 | 17.35 | 120.6 | 105.88 | 4.02 | 0.93 |
| NT | 120.895 | 31.927 | 0 | — | 0.00 | 15.36 | 8.16 | 194.02 | 1.25 | 1.31 | 113.29 | 101.23 | 4.49 | 0.92 |

| | | | | | | | | | | | | | | |
|---|---|---|---|---|---|---|---|---|---|---|---|---|---|---|
| C1 | 121.089 | 31.747 | 0 | 0-2 cm | 0.17 | 14.25 | 8.01 | 200.92 | 2.11 | 0.95 | 112.01 | 97.93 | 5.19 | 0.68 |
| C2 | 121.202 | 31.665 | 0 | 0-2 cm | 0.17 | 13.96 | 8.08 | 200.08 | 2.05 | 0.79 | 113.12 | 96.93 | 4.07 | 0.49 |
| C3 | 121.320 | 31.577 | 0 | — | 0.17 | 12.99 | 8.12 | 199.29 | 2.01 | 1.61 | 113.87 | 96.7 | 3.11 | 0.51 |
| C4 | 121.427 | 31.506 | 0 | 0-2 cm | 0.17 | 12.70 | 8.12 | 200.05 | 1.50 | 1.64 | 94.67 | 98.6 | 1.03 | 0.73 |
| C5 | 121.534 | 31.418 | 0 | 0-2 cm | 0.17 | 12.87 | 8.20 | 209.16 | 1.32 | 1.96 | 117.24 | 95.76 | 0.21 | 0.62 |
| C6 | 121.652 | 31.329 | 0 | 0-2 cm | 0.21 | 13.05 | 8.33 | 191.84 | 1.71 | 0.79 | 122.56 | 93.51 | 0.14 | 0.42 |
| C7 | 121.777 | 31.241 | 0 | 0-2 cm | 0.41 | 13.11 | 8.13 | 194.02 | 1.58 | 1.24 | 151.17 | 88.64 | 1.09 | 0.81 |
| C8 | 121.883 | 31.159 | 0 | 0-2 cm | 5.09 | 13.31 | 8.19 | 211.53 | 0.74 | 1.54 | 129.94 | 79.14 | 0.37 | 1.04 |
| C9 | 121.968 | 31.071 | 0 | 0-2 cm | 10.71 | 14.18 | 8.09 | 212.11 | 0.83 | 1.51 | 107.41 | 71.74 | 0.22 | 0.99 |
| C1_B | 121.089 | 31.747 | 10 | — | 0.17 | 14.33 | 8.08 | 202.50 | 1.69 | 0.82 | 111.5 | 96.16 | 5.16 | 0.74 |
| C2_B | 121.202 | 31.665 | 12 | — | 0.17 | 14.44 | 8.10 | 198.22 | 1.76 | 2.27 | 112.73 | 90.25 | 4.03 | 0.78 |
| C3_B | 121.320 | 31.577 | 15 | — | 0.17 | 14.11 | 8.09 | 206.23 | 1.94 | 2.62 | 112.73 | 98.68 | 3.12 | 0.79 |
| C4_B | 121.427 | 31.506 | 12 | — | 0.17 | 13.89 | 8.13 | 208.45 | 1.99 | 2.23 | 91.86 | 86.83 | 0.58 | 0.86 |
| C5_B | 121.534 | 31.418 | 7 | — | — | — | — | — | — | — | — | — | — | — |
| C6_B | 121.652 | 31.329 | 6 | — | 0.24 | 13.00 | 8.21 | 200.52 | 1.60 | 2.11 | 126.00 | 84.79 | 0.09 | 0.76 |
| C7_B | 121.777 | 31.241 | 10 | — | 2.65 | 13.11 | 8.12 | 197.73 | 1.14 | 2.01 | 139.73 | 88.47 | 0.55 | 0.99 |
| C8_B | 121.883 | 31.159 | 5 | — | 11.16 | 13.47 | 8.13 | 206.52 | 1.37 | 1.70 | 105.12 | 71.42 | 0.23 | 1.09 |
| A1 | 122.044 | 30.988 | 0 | — | 16.25 | 14.24 | 8.20 | 202.75 | 0.95 | 1.14 | 60.78 | 55.33 | 0.17 | 0.75 |
| A2 | 122.233 | 30.947 | 0 | 0-2 cm | 16.58 | 14.26 | 8.12 | 154.30 | 0.86 | 1.05 | 70.62 | 52.55 | 0.22 | 0.88 |
| A3 | 122.381 | 30.906 | 0 | 0-2 cm | 12.96 | 13.93 | 8.07 | 210.03 | 4.07 | 0.74 | 86.19 | 64.77 | 0.26 | 0.87 |
| A4 | 122.500 | 30.871 | 0 | 0-2 cm | 17.10 | 14.56 | 8.15 | 206.82 | 5.46 | 0.63 | 68.6 | 52.34 | 0.30 | 0.77 |
| A5 | 122.648 | 30.829 | 0 | 0-2 cm | 21.91 | 15.08 | 8.16 | 207.32 | 3.61 | 0.44 | 51.5 | 41.02 | 0.29 | 0.70 |
| A6 | 122.802 | 30.777 | 0 | 0-2 cm | 30.03 | 14.81 | 8.21 | 194.05 | 0.69 | 0.28 | 17.81 | 15.26 | 0.32 | 0.32 |
| A7 | 122.998 | 30.718 | 0 | 0-2 cm | 32.95 | 15.79 | 8.28 | 202.04 | 3.65 | 0.13 | 5.9 | 7.79 | 0.26 | 0.15 |

For Review Only

| | | | | | | | | | | | | | | |
|---|---|---|---|---|---|---|---|---|---|---|---|---|---|---|
| A8 | 123.247 | 30.641 | 0 | 0-2 cm | 33.54 | 15.87 | 8.35 | 207.97 | 3.37 | 0.00 | 0.31 | 0.94 | 0.07 | 0.01 |
| A10 | 123.750 | 30.500 | 0 | — | 33.72 | 14.59 | 8.36 | 200.67 | 2.91 | 0.07 | 0.1 | 0.74 | 0.05 | 0.02 |
| A2_B | 122.233 | 30.947 | 5 | — | 19.79 | 13.95 | 8.13 | 209.15 | 0.94 | 0.97 | 65.62 | 48.93 | 0.23 | 0.85 |
| A3_B | 122.381 | 30.906 | 10 | — | 27.37 | 14.30 | 8.07 | 186.60 | 3.24 | 2.09 | 31.14 | 26.21 | 0.11 | 0.75 |
| A4_B | 122.500 | 30.871 | 16 | — | 31.17 | 15.13 | 8.18 | 193.88 | 2.37 | 1.54 | 14.84 | 13.12 | 0.25 | 0.28 |
| D1 | 122.016 | 31.342 | 0 | — | 29.88 | 12.81 | 8.10 | 195.33 | 1.94 | 0.71 | 112.92 | 84.28 | 0.23 | 0.87 |
| D2 | 122.224 | 31.528 | 0 | 0-2 cm | 31.25 | 11.91 | 8.11 | 125.90 | 1.97 | 0.65 | 21.26 | 19.61 | 0.33 | 0.54 |
| D3 | 122.411 | 31.699 | 0 | 0-2 cm | 31.28 | 11.74 | 8.11 | 103.29 | 0.99 | 0.06 | 12.33 | 13.4 | 0.21 | 0.41 |
| D4 | 122.592 | 31.838 | 0 | 0-2 cm | 31.33 | 11.84 | 8.14 | 88.36 | 0.90 | 0.42 | 13.30 | 14.19 | 0.34 | 0.47 |
| D5 | 122.774 | 31.987 | 0 | — | 31.25 | 12.01 | 8.18 | 166.46 | 0.36 | 2.63 | 12.78 | 15.2 | 0.23 | 0.41 |
| D6 | 122.993 | 32.169 | 0 | — | 31.56 | 11.83 | 8.14 | 157.72 | 0.50 | 0.22 | 12.74 | 14.48 | 0.29 | 0.41 |
| D7 | 123.212 | 32.356 | 0 | — | 32.13 | 11.85 | 8.15 | 194.38 | 0.82 | 0.00 | 11.89 | 16.07 | 0.20 | 0.54 |
| D8 | 123.409 | 32.537 | 0 | 0-2 cm | 31.95 | 11.27 | 8.14 | 176.62 | 1.12 | 0.38 | 11.86 | 16.79 | 0.15 | 0.58 |
| D9 | 123.623 | 32.687 | 0 | 0-2 cm | 32.28 | 10.85 | 8.12 | 193.90 | 1.43 | 0.00 | 10.73 | 15.85 | 0.17 | 0.50 |
| D10 | 123.804 | 32.852 | 0 | 0-2 cm | 32.99 | 11.17 | 8.15 | 190.50 | 0.82 | 0.10 | 6.62 | 11.15 | 0.22 | 0.40 |
| D11 | 124.002 | 33.007 | 0 | 0-2 cm | 33.41 | 11.34 | 8.15 | 188.30 | 2.29 | 0.39 | 3.92 | 11.24 | 0.14 | 0.28 |
| D12 | 124.189 | 33.167 | 0 | 0-2 cm | 33.54 | 11.10 | 8.16 | 98.53 | 2.26 | 0.28 | 3.91 | 10.92 | 0.29 | 0.27 |
| D2_B | 122.224 | 31.529 | 12 | — | 31.27 | 11.83 | 8.08 | 114.60 | 1.74 | 0.47 | 21.08 | 19.52 | 0.29 | 0.54 |
| D6_B | 122.993 | 32.169 | 30 | — | 32.12 | 11.86 | 8.36 | 186.05 | 0.95 | 0.15 | 12.69 | 15.11 | 0.32 | 0.47 |
| D10_B | 123.804 | 32.852 | 38 | — | 33.09 | 12.19 | 8.15 | 198.34 | 1.10 | 0.31 | 7.02 | 11.12 | 0.27 | 0.46 |
| D12_B | 124.189 | 33.1668 | 58 | — | 32.76 | 12.20 | 8.21 | 202.71 | 0.40 | 0.14 | 7.28 | 11.29 | 0.22 | 0.51 |

**Note:** The salinity of the freshwater region has not been measured and is recorded as 0. B represents the bottom water. "—" means there is no sample. The stations between Zhu Tuo (ZT) and Nan Jing (NJ) represent the freshwater region (FR); stations from Nan Tong (NT) to C9 represent the transition region (TR); stations from A1 to A10 and from D1 to D12 represent the coastal region (CR). BB, Bei Bei; XK, Xia Kou; WL, Wu Long; FD, Feng Du; ZX, Zhong Xian; WZ, Wan Zhou; YY, Yun Yang; FJ, Feng Jie; DR, Daning River; XX, Xiang Xi; ZG, Zi Gui; YC, Yi Chang; YY, Yue Yang; WH, Wu Han; JJ, Jiu Jiang; HK, Hu Kou; DT, Da Tong.

For Review Only

**Table S2** PERMANOVA analyzed the differences between regional communities among different habitats (water [W] and sediment [S]) and lifestyles (free-living [F] and particle-associated [P]).

| Group | Variation ($R^2$) | P |
|---|---|---|
| FR-W/FR-S | 0.269 | 0.001 |
| FR-W/TR-W | 0.160 | 0.001 |
| FR-W/CR-W | 0.235 | 0.001 |
| FR-S/TR-S | 0.223 | 0.001 |
| FR-S/CR-S | 0.362 | 0.001 |
| TR-W/TR-S | 0.322 | 0.001 |
| TR-W/CR-W | 0.308 | 0.001 |
| TR-S/CR-S | 0.204 | 0.001 |
| CR-W/CR-S | 0.166 | 0.001 |
| FR-F/TR-F | 0.217 | 0.001 |
| FR-F/CR-F | 0.369 | 0.001 |
| FR-P/TR-P | 0.238 | 0.001 |
| FR-P/CR-P | 0.214 | 0.001 |
| FR-F/FR-P | 0.166 | 0.001 |
| TR-F/CR-F | 0.340 | 0.001 |
| TR-P/CR-P | 0.331 | 0.001 |
| TR-F/TR-P | 0.117 | 0.001 |
| CR-F/CR-P | 0.074 | 0.001 |

For Review Only

**Table S3** Comparison of spatial differences among various habitats and lifestyle communities based on the PERMANOVA analysis.

| Group (FR *vs* TR *vs* CR) | Variation |
|---|---|
| Water | $R^2 = 0.318$, $p = 0.001$ |
| Sediment | $R^2 = 0.339$, $p = 0.001$ |
| FL | $R^2 = 0.416$, $p = 0.001$ |
| PA | $R^2 = 0.328$, $p = 0.001$ |

**Table S4** Spearman's correlations of the microbial community with environmental factors based on Mantel tests and partial Mantel tests.

| Factors | Mantel tests | | | partial Mantel tests | |
|---|---|---|---|---|---|
| | **Total** | **FL** | **PA** | **FL** | **PA** |
| Sal | 0.734*** | 0.815*** | 0.721*** | 0.790*** | 0.695*** |
| Temp | 0.430*** | 0.458*** | 0.406*** | 0.413*** | 0.336*** |
| pH | 0.140*** | 0.081 | 0.182** | 0.019 | 0.095* |
| DO | 0.260*** | 0.258*** | 0.201*** | 0.299*** | 0.228*** |
| Chl *a* | 0.111** | 0.113* | 0.093* | 0.087* | 0.053 |
| $NH_4^+$ | 0.369*** | 0.359*** | 0.376*** | 0.243*** | 0.169*** |
| $NO_3^-$ | 0.761*** | 0.823*** | 0.719*** | 0.833*** | 0.767*** |
| $SiO_3^{2-}$ | 0.790*** | 0.860*** | 0.752*** | 0.867*** | 0.796*** |
| $NO_2^-$ | 0.204*** | 0.158** | 0.262*** | 0.077 | 0.149** |
| $PO_4^{3-}$ | 0.336*** | 0.343*** | 0.324*** | 0.346*** | 0.349*** |

The significances are tested based on 999 permutations, and bold values indicate significant correlation relationship. ***, $p$ < 0.001, **, $p$ < 0.01, *, $p$ < 0.05). Sal, salinity; Temp, temperature.

**Table S5** Network topological parameters for different lifestyle communities.

| **Topological parameters** | **FL** | **PA** |
|---|---|---|
| Edges | 7689 | 11583 |
| Nodes | 404 | 656 |
| Clustering coefficient | 0.621 | 0.547 |
| Average path length | 3.124 | 3.546 |
| Diameter | 12 | 10 |
| Graph density | 0.094 | 0.054 |
| Modularity | 0.303 | 0.534 |
| Average degree | 38.064 | 35.314 |
| Betweenness centralization | 0.099 | 0.184 |
| Degree centralization | 0.260 | 0.145 |

For Review Only

**Table S6** Network topological parameters for different communities.

| Topological parameters | FR | | TR | | CR | |
|---|---|---|---|---|---|---|
| | FL | PA | FL | PA | FL | PA |
| Edges | 233 | 484 | 537 | 2154 | 1957 | 5228 |
| Nodes | 170 | 265 | 136 | 280 | 218 | 472 |
| Clustering coefficient | 0.394 | 0.432 | 0.841 | 0.659 | 0.665 | 0.630 |
| Average path length | 5.815 | 3.672 | 3.538 | 3.909 | 1.926 | 2.956 |
| Diameter | 17 | 12 | 12 | 15 | 8 | 11 |
| Graph density | 0.016 | 0.014 | 0.058 | 0.055 | 0.083 | 0.047 |
| Modularity | 0.794 | 0.678 | 0.315 | 0.294 | 0.293 | 0.186 |
| Average degree | 2.741 | 3.653 | 7.897 | 15.386 | 17.954 | 22.153 |
| Betweenness centralization | 0.096 | 0.031 | 0.068 | 0.073 | 0.011 | 0.016 |
| Degree centralization | 0.061 | 0.073 | 0.164 | 0.185 | 0.263 | 0.184 |

For Review Only
